# Nutrition and Metabolism of Minerals in Fish

**DOI:** 10.3390/ani11092711

**Published:** 2021-09-16

**Authors:** Santosh P. Lall, Sadasivam J. Kaushik

**Affiliations:** 1 National Research Council of Canada, Halifax, NS B3H 3Z1, Canada; 2 Retd. INRA, 64310 St Pée sur Nivelle, France; 3 Ecoaqua Institute, Universidad de Las Palmas de Gran Canaria, 35214 Las Palmas, Spain

**Keywords:** minerals, trace elements, fish, copper, iron, selenium, manganese, zinc, calcium, phosphorus, magnesium

## Abstract

**Simple Summary:**

Our aim is to introduce the mineral nutrition of fish and explain the complexity of determining requirements for these elements, which are absorbed and excreted by the fish into the surrounding water. To date, only the requirements for nine minerals have been investigated. The review is focused on the absorption and the dietary factors that reduce their absorption from feed ingredients of plant and animal origin. Some diseases, such as cataracts, anemia and bone deformity, have been linked to dietary deficiency of minerals.

**Abstract:**

Aquatic animals have unique physiological mechanisms to absorb and retain minerals from their diets and water. Research and development in the area of mineral nutrition of farmed fish and crustaceans have been relatively slow and major gaps exist in the knowledge of trace element requirements, physiological functions and bioavailability from feed ingredients. Quantitative dietary requirements have been reported for three macroelements (calcium, phosphorus and magnesium) and six trace minerals (zinc, iron, copper, manganese, iodine and selenium) for selected fish species. Mineral deficiency signs in fish include reduced bone mineralization, anorexia, lens cataracts (zinc), skeletal deformities (phosphorus, magnesium, zinc), fin erosion (copper, zinc), nephrocalcinosis (magnesium deficiency, selenium toxicity), thyroid hyperplasia (iodine), muscular dystrophy (selenium) and hypochromic microcytic anemia (iron). An excessive intake of minerals from either diet or gill uptake causes toxicity and therefore a fine balance between mineral deficiency and toxicity is vital for aquatic organisms to maintain their homeostasis, either through increased absorption or excretion. Release of minerals from uneaten or undigested feed and from urinary excretion can cause eutrophication of natural waters, which requires additional consideration in feed formulation. The current knowledge in mineral nutrition of fish is briefly reviewed.

## 1. Introduction

All aquatic animals require minerals for their vital physiological and biochemical functions and to maintain their normal life processes. Fish live in a wide range of salinity levels (0–35 0/00) in freshwater (FW), seawater (SW) and brackish water (BW) environments, and, unlike other vertebrates, absorb minerals from the diet as well as the surrounding water. Most of the essential minerals required for animals and other vertebrates [1] have been detected in fish tissues. The essentiality of macrominerals (calcium, phosphorus, magnesium, sodium, potassium and chloride) and certain trace elements (cobalt, copper, iodine, iron, manganese selenium and zinc) have been confirmed in fish [2,3]. Other trace elements (arsenic, boron, chromium, fluorine, nickel, lithium, lead, molybdenum, silicon and vanadium) considered essential for humans and animals based on the impairment of specific physiological functions have not been reported in fish. Mineral nutrition of fish has received limited attention as compared to other nutrients. The main focus of the current review is trace element nutrition and metabolism of fish with a brief discussion on major macrominerals. A significant amount of research has been directed towards the physiological aspects of waterborne mineral toxicity and is the subject of comprehensive reviews [4,5].

The biochemical mechanisms of mineral metabolism in fish are generally considered similar to those of terrestrial animals at the cellular level. The exchange of ions from across the gills and skin of fish in surrounding water complicates the determination of the quantitative dietary requirement [6]. Gills comprise over 50% of the surface area of the fish and are considered the major route of uptake of waterborne minerals in FW. In SW, fish exhibit obligatory drinking as part of their overall physiological osmoregulatory mechanism to maintain internal body fluids substantially hypotonic to the external salinity of seawater [7]. Therefore, absorption of dietary minerals from the gastrointestinal tract is important in FW fish, whereas in SW fish, both dietary and waterborne minerals are absorbed. The life cycle of anadromous fish like salmonids consists of FW and SW, where they gradually adapt to a marine environment and acquire inorganic elements by drinking SW, similar to marine fish. 

In the past two decades, the number of farmed crustaceans, FW and marine fish species has increased considerably [8]. Often a generalization is made estimating their mineral requirements based on salmonids and certain freshwater species with proper consideration for the determination made on different types of diets (purified, semi-purified and practical diet) and the use of inorganic supplements with high bioavailability. In addition, fish feeds increasingly comprise alternate sources of plant-based feed ingredients as compared to previously widely used fish meal due to the increase in global aquaculture production and its limited supply. Obviously, there is a need to reassess the mineral requirements as well as their bioavailability from a wide range of plant feed ingredients as well as new alternate sources of feed ingredients compared to fish meal. The absorption of minerals may vary among fishes because of differences in gastric acid secretion in fish with stomach and agastric or stomachless fish [6] as well as uptake of minerals from water. There are also differences in the methodology used to measure the mineral requirements and response criteria (e.g., growth, feed utilization, whole body and/or vertebrae, plasma/serum and tissue mineral concentration, hematology and changes in specific enzyme activities).

Minerals are known to interact with other nutrients due to their lability and tendency to form chemical bonds. The term “interaction” for minerals was defined by O’Dell [9] as “interrelationships among mineral elements as revealed by physiological or biochemical consequences”. Such interactions are broadly classified as positive or synergistic, or negative or antagonistic. Direct positive interactions between elements in structural processes such as the requirement of copper (Cu) and iron (Fe) for hemoglobin formation, calcium (Ca), phosphorus (P) and magnesium (Mg) for formation of bone hydroxyapatite and an interaction of Mn with Zn for the proper conformational shape of RNA molecules in the liver have been widely recognized. Antagonistic relationships are considered to occur when trace elements with a similar electronic configuration and ionic radius compete for binding sites, such as zinc (Zn) and cadmium (Cd) in metallothionein, and Mg/manganese (Mn) substitutions at active sites of enzymes. In the gastrointestinal tract, antagonistic relationships may occur by a simple mechanism, which involves a chemical reaction forming an insoluble complex between minerals such as Cu and sulfur (S) to form copper sulfide or a mineral and another dietary component such as Zn combined with phytic acid to form phytate [10]. A range of potential mineral–mineral and mineral–vitamin interactions have also been reported in fish [11]. Specific trace element interactions are discussed in appropriate individual sections.

Extensive research conducted on animals has clearly shown that mineral requirements are significantly affected by their bioavailability from different forms of feed supplements and feed ingredients. Bioavailability is defined as the proportion of dietary intake of an element that is utilized for biochemical or physiological functions [9]. Generally, the bioavailability reflects the absorption of the nutrient. From a practical standpoint, measurement of biochemical and physiological functions may not always be possible. Ammerman [12] suggested that more emphasis should be on the degree to which an ingested element could be utilized for metabolism. Limited information exists on the utilization and metabolism of trace elements in fish. The following methods have been used to determine the bioavailability of trace minerals in animals [9,12,13]: (a), growth, (b) apparent absorption or digestibility, (c) plasma or tissue mineral concentration, (d) mineral retention or balance, (e) specific enzyme activity (e.g., superoxide dismutase, glutathione peroxidase) or blood parameter (e.g., hemoglobin level), (f) prevention of deficiency signs. Some of these methods have also been used in fish to estimate the bioavailability of Zn, Fe and selenium (Se) from chemically defined or practical diets based on fish meal and plant protein and are discussed later in individual sections. 

Aquatic animals accumulate excessive minerals from water which can interact with as well as inhibit proteins that facilitate essential ion transport [14,15,16]. Metal binding reactions include competition at the biotic ligand with other cations (e.g., Na^+^, H^+^, Ca^2+^) and competing complexation reactions for the metal by other ligands in solution including inorganic anions (e.g., chloride, hydroxide, sulfide) as well as organic ligands such as dissolved organic carbon [16]. For example, accumulation of Cu at the fish gill interferes with Na^+^ uptake, thereby disrupting ionic balance in the organism, leading to toxicity [17]. Similarly, Cd and Zn interfere with Ca uptake at the fish gill, leading to hypocalcemia and toxicity [18]. The ability of fish to regulate high concentrations of trace elements originating from water varies among different fish species. The major route of uptake for some metals, such as Se, mercury (Hg) and arsenic (As), is trophic transfer through the food chain. Certain fish are able to excrete high proportions of excessive metal intake and consequently regulate the concentration in their body at relatively normal levels [19]. The soluble trace elements in water are considered more toxic than higher dietary intake of minerals such as Cu, Fe and Zn. The subject of aquatic toxicology to fish is beyond the scope of this review. 

Minerals discharged from uneaten feeds, excretion of undigested material in feces and urine from hatcheries and aquaculture operations, directly influence the aquatic environment [20]. These minerals excreted in soluble and particulate forms affect water quality. The particulate form can settle to the bottom of ponds or tanks or accumulate at the end of raceways or in sediment under fish cages. The chemical composition of feedstuffs and type of inorganic or organic mineral supplements used have significant effects on the breakdown of organically bound minerals in feces and the amount of soluble inorganic compounds from urine in water. Microorganisms and several environmental factors (e.g., water current, temperature, dissolved oxygen levels, pH and salinity) and the type of microorganisms also affect minerals released from feces and urine in natural waters. Experimental studies have demonstrated that elevated concentrations of Zn and Cu originating from feeds in sediments under sea cages decrease to background levels after chemical remediation [21,22]. Some of these trace elements (e.g., Cu, Zn, Cd, etc.) can also be associated with naturally occurring organic debris. Organic particles associated with metals are remobilized into the dissolved phase, and soluble Cu, Zn and Cd form complexes with organic ligands in pore waters [23,24]. Fishery by-products and other ingredients may contribute to a minor amount of Cd in the finished feed. Cadmium can be scavenged from the water column and ultimately deposited with organic particles [25,26]. Several countries have developed legal measures to regulate the maximum limit of mineral supplementation in fish feeds to minimize their impacts on the environment.

The main purpose of this review is to update current knowledge of mineral nutrition of fish and to identify areas that require future research, particularly trace elements.

## 2. Microminerals

Optimum levels of essential macro- and microminerals are required for growth and maintenance of normal health of farmed fish. Four broad biochemical functions of micro- or trace elements are widely recognized: (a) catalytic, (b) structural, (c) physiological and (d) regulatory [27]. Trace minerals can act as catalysts in enzyme and endocrine systems, as integral and specific components of the structure of metalloenzymes and hormones or as activators (coenzymes) within those systems. More than one-third of all proteins require a trace element cofactor for normal function [28,29]. Numerous metalloenzymes are required for a wide range of metabolic activities such as energy production, protein digestion, cell replication and antioxidant activity, which are discussed in later sections. Deficiencies or suboptimum levels of the trace element may cause a decrease in or loss of enzyme activities [30]. Increased attention has been focused on certain micronutrients and immunostimulants to reduce susceptibility to various stressors and diseases, as well as enhance the overall health of humans, animals and fish [31,32,33]. The concept is based on understanding the contribution of minerals in reducing the detrimental effects of free radicals and toxic metabolites on immune processes in the animal’s body. The knowledge in this area on the role of minerals in fish or shrimp as compared to animals and humans is scant.

### 2.1. Copper

It is widely accepted that Cu is an essential trace element required for cellular functioning of all living organisms. Copper ions have a unique chemistry due to their ability to adopt distinct redox states, either oxidized Cu^2+^ or the reduced state Cu^3+^. Due to the high avidity of biological ligands, free ionic Cu^2+^ is present in physiological fluids at extremely low concentrations. Copper metalloenzymes are involved in Fe metabolism, cellular energy production (cytochrome c oxidase), protection of cells from free radical damage (superoxide dismutase), collagen synthesis (lysyl oxidase) in brain neurotransmitters (dopamine hydroxylase and peptidyl alpha amidating monoxygenase) and melanin production (tyrosinase) [34]. Four Cu-containing enzymes, known as multi-copper oxidases (MCO) or ferroxidases, oxidize Fe^2+^ to Fe^3+^ ion, the form of iron that can be incorporated onto the protein transferrin for transport to the site of red blood cell formation. The MCO family comprises circulating and membrane-bound ceruloplasmin and two other proteins [35]. Metabolic changes in Cu-requiring proteins or alterations in enzyme activities may cause pathophysiological conditions [36].

Fish absorb Cu via the gills and digestive tract; however, the diet is considered a major source of Cu for growth, development and essential physiological functions [14,37]. The gill may contribute to a significant amount of Cu uptake depending upon the Cu concentration of the water [38], particularly when dietary Cu intake is low [39]. In rainbow trout fed a Cu-deficient diet, waterborne Cu uptake contributed the major proportion (60 %) of the body’s Cu; however, feeding high levels of Cu contributed to nearly 99 % of body Cu [37]. Uptake of Cu from water and its toxicity have been extensively studied and reviewed [17,40]. Copper uptake is facilitated via two distinctive mechanisms: (a) by a transmembrane protein (Cu transporter 1) and (b) the apical Na^+^ uptake pathways located at branchial epithelial cells. The former pathway is insensitive to external Cu concentrations, and the latter pathway involving Na^+^ uptake is sensitive to Cu [41]. In gastrointestinal Cu transport, apical Cu uptake appears to be passive while basolateral transport is active and rate limiting [42,43]. Copper is known to induce oxidative stress, olfactory impairment, increased plasma ammonia and disturbed acid–base balance [17,44].

Maintaining Cu homeostasis demands a critical physiological orchestration between Cu uptake and distribution within cells, and detoxification and removal. In fish and terrestrial vertebrates, the liver is the main organ involved in Cu homeostasis and metabolism [41,45]. Most studies on Cu homeostasis in fish have been undertaken in FW fish. After gill uptake and absorption from the gut, Cu is cleared from blood by the liver and incorporated into ceruloplasmin for transport to extrahepatic organs, stored in Cu–protein complexes or excreted via the bile [19,46].

#### 2.1.1. Requirement

The dietary Cu requirement has been reported for several freshwater and marine fish (Table 1). These requirements are relatively low as compared to other trace elements. It is necessary to know the concentration of Cu in water, feed ingredients and tissue prior to requirement studies, in order to properly estimate the quantitative Cu requirement [1]. Copper in the water alone cannot meet the requirements so oral administration of Cu is essential for aquatic animals [3]. The dietary Cu requirement would depend on the physiological status of the animal, the concentration of Cu in the water and also the levels of elements that are metabolic antagonists of copper, such as Fe, Zn, Cd and Mo [1,47]. A meta-analysis of published information on the Cu requirement of several fish species showed relatively close estimates for the Cu requirement (mg kg^−1^ diet) based on the following parameters: weight gain (WG), [6,48] whole body, liver or vertebral Cu concentration [6,48] and liver CuZn SOD (superoxide dismutase)) activity [6,48]. The antagonistic effects of Zn and Cu have not been observed in rainbow trout [49]. An interaction between dietary Cu and Se was observed in Atlantic salmon, where liver Se was inversely related to dietary Cu concentration [50]. 

#### 2.1.2. Deficiency 

Copper deficiency is well recognized in domestic animals [1]; however, gross deficiency signs of Cu have not yet been reported in fish. Unlike terrestrial vertebrates, small amounts of Cu are supplied by water as well as feeds. Therefore, overt deficiency signs would only occur during a long period of Cu deprivation. To date, the response criteria used to detect the Cu deficiency and adequacy of this element are not consistent, as shown in studies conducted on 14 FW and marine fish species (Table 1). A decrease in growth, body and liver Cu concentration and ceruloplasmin and enzyme activities (liver CuZn SOD, heart cytochrome c oxidase) have been reported. In a long-term study, carp fed diets containing white fish meal without any supplementary Cu were found to exhibit reduced growth and cataract formation [73]. Other effects may include low pigmentation of the embryo, spinal cord deformation, cranial malformations, jaw underdevelopment, decreased length, increased time to complete yolk absorption, edema and opaque yolk sacs [74]. Low concentrations of waterborne Cu affected the hatching of fish eggs by inactivating chorionase activity, which causes osmotic disturbances and also affects the muscular movements necessary to break the eggshell [74,75].

#### 2.1.3. Toxicity

Generally, dietary Cu toxicity rarely occurs under practical feeding conditions except when there is an error in feed mixing or the use of Cu-contaminated feed ingredients. Toxicity of Cu has been experimentally produced in rainbow trout, Atlantic salmon, rockfish (Sebastes schlegeli), Nile tilapia and African walking catfish (Clarias gariepinus), however, the tolerance of Cu varies among fish species [76,77,78,79,80,81,82,83,84,85,86]. Signs of oral Cu toxicity include reduced growth and feed utilization, changes in hematological parameters and tissue lipid peroxidation. Dietary copper toxicity measured as growth inhibition in rainbow trout was approximately 664–730 mg Cu kg^−1^ feed corresponding to 44 mg Cu kg^−1^ body weight per day [76,80]. Much lower concentrations of Cu (34 mg kg^−1^) in Atlantic salmon diet caused tissue lipid peroxidation [87]. The estimated threshold of diet-borne Cu toxicity for Atlantic salmon parr and fry, based on organ copper burden and reduced growth, ranged from 15 to 17 mg Cu kg^−1^ per day [80]. The EU Commission-authorized maximum content of Cu in complete feed for salmonids and other fish species is 25 mg kg^−1^ [88].

Aquatic Cu toxicity in fish and other organisms has been the subject of extensive research for five decades [17] and is briefly mentioned in this section. The mechanism of Cu toxicity differs in FW and SW. In FW, Cu toxicity varies with water hardness, pH, anions and dissolved organic carbon. Copper toxicity is more lethal in soft compared to hard waters rich in cations (e.g., Ca^2+^ and Mg^2+^) as cations reduce bioavailability of Cu and thus accentuate toxic effects [89]. Copper is more toxic under acidic conditions (pH < 6). Anions and dissolved organic carbon bind to Cu and form compounds, thus reducing the toxic effects [90]. In SW, waterborne Cu toxicity affects not only gills but also the intestine because marine fish drink to compensate for water loss into the surrounding water. The cause of reduced oxidative metabolism appears to be gill damage, manifested as either disruption of branchial structure, secretion of mucus that binds metals and impedes the rates of diffusion, inhibition of respiratory enzymes and damage to gill oxygen receptors [17]. In addition to gills, Cu toxicity also affects the liver, kidney, heart, brain and reproductive organs. In summary, exposure to higher levels of Cu in water affects several physiological and biochemical functions, affecting the performance of the whole organism by decreasing metabolic rate and causing oxidative stress [40,91]. Oxidative damage from Cu exposure has been observed as changes in biomarkers (e.g., protein carbonyls, lipid peroxidation and DNA damage products) in the gill, liver and intestine of numerous fish species [40]. 

#### 2.1.4. Bioavailability

Limited information exists on the availability of Cu from feed ingredients or Cu feed additives. Feed ingredients of plant and animal sources show a variable Cu content resulting from either contaminants originating from soil or during the processing of plant and animal products. Common Cu feed additives commercially available to supplement fish and animal feeds are copper sulfate; cupric chloride; cupric carbonate; cupric acetate; cupric methionate; cupric oxide; cupric chelate of amino acid hydrate; copper lysine sulfate; cupric chelate of glycine hydrate; copper chelate of hydroxy analogue of methionine; dicopper chloride trihydroxide and copper bislysinate. In most fish studies, copper sulfate has been used as a dietary supplement or a standard for the evaluation of Cu bioavailability. To date, limited research effort has been made to measure the bioavailability of Cu from inorganic and organic forms or the residual Cu present in feed ingredients. Some studies dealing with the inclusion of different forms of trace elements such as Zn, Mn and Se in plant protein-rich diets suggest that dietary minerals supplemented in the organic form could be reasonably considered more effective than the inorganic and encapsulated forms of supply [92]. A wide range of methods (e.g., plasma/liver Cu, erythrocyte SOD activity, plasma ceruloplasmin and bile Cu) have been used to estimate Cu bioavailability in animals [13]. 

Studies conducted on bioavailability from different organic Cu sources in animal feeds show similar bioavailability while others show higher bioavailability relative to copper sulfate NRC [47]. Some of the differences have been attributed to species differences, age of animals, response criteria and the method used. In rainbow trout fed semi-purified diets, cupric sulfate Cu-proteinate and Cu-lysine showed similar bioavailability [93]. Cu-Met and nano-copper oxide were also shown to be more bioavailable than copper sulfate in Russian sturgeon [65]. Metal ion interactions with Cu-Zn have been observed in higher animals [94], but no significant antagonism between Cu and Zn was observed in rainbow trout fed plant-based diets [95]. 

### 2.2. Iron

Iron is one of the most investigated essential trace elements, and is present in all body cells of vertebrates [3,96]. It is essential for the functioning of several biochemical processes, which include the electron transfer reaction, gene regulation, binding and transport of oxygen and regulation of cell growth and differentiation. The most important Fe-containing compounds are the heme proteins, hemoglobin, myoglobin and cytochromes. Enzymes containing non-heme Fe such as iron–sulfur cluster proteins (e.g., nicotinamide adenine dinucleotide (NADH) dehydrogenase, succinate dehydrogenase, xanthine oxidase) are involved in energy metabolism. Another group of Fe-containing enzymes (e.g., hydrogen peroxidases) are well known to act on reactive molecules originating as the by-products of oxygen metabolism. The formation of the reduced form of iron, Fe^2+^, can produce highly reactive hydroxyl and lipid radicals, which can damage lipid membranes, nucleic acids and proteins. In mammals, iron homeostasis involves the regulation of its absorption into the body, regulation of iron entry into cells, the incorporation of iron into proteins, the storage of iron in ferritin and the regulation of iron released for transport to other cells and organs. 

Although there is relatively little information on absorption and metabolism of iron in fish, some studies suggest that mechanisms of iron absorption from the digestive tract and storage and excretion may be similar to those in other vertebrates [14,97]. It is widely recognized that Fe metabolism involves its absorption from the gastrointestinal tract into the body, entry into cells, the incorporation into proteins and the storage of Fe in ferritin in different tissues. Although fish gills play an important role in Fe acquisition, the uptake is relatively low [98,99]. Like other trace elements, the gastrointestinal tract is considered the major route of Fe absorption [100]. The uptake of Fe from natural waters is considered low. Fish gills play an important role in Fe acquisition [101], particularly in developing fish when the digestive tract is not fully functional [98]. The gastrointestinal tract is considered the major site of Fe absorption [99]. In the acidic environment of the stomach, ferric ion is released from the ingested food materials and binds to mucin which may facilitate metal solubility in the small intestine of fish [100]. It is proposed that epithelial mucus secretion may play an important role in maintaining metal solubility in fish [102,103]. The mechanism of Fe absorption in marine fish is not clear. They ingest significant amounts of Ca and Mg from SW and also secrete large quantities of bicarbonate [104] that could potentially cause problems for intestinal iron uptake. It has been suggested that metal–mucus chelates that retain the metal in solution in the intestinal tract, reducing agents in foods (e.g., ascorbic acid) and other physiological factors in the gut may modify Fe solubility and enhance its absorption [14,98]. Food is regarded as the main source of Fe for metabolic purposes [14,98]. A small amount of Fe is eliminated via the liver (i.e., bile) and to some extent by the kidney [102]. 

#### 2.2.1. Requirement

The Fe requirement reported for certain fish species (Table 1) ranges from 30–170 mg kg^−1^ diet with the exception of gibel carp which has a reported optimum requirement value of 202 mg kg^−1^ diet. Response criteria used to estimate Fe requirements were weight gain, whole body, liver and plasma iron concentration and hematological parameters (hemoglobin, hematocrit, mean corpuscular volume, mean corpuscular hemoglobin). A meta-analysis of published information on Fe requirements of several fish species showed estimates for Fe requirements ranging from 58.8–166.4 mg kg^−1^ with a wide variation for all parameters tested [105]. It should be emphasized that weight gain alone may not provide a good estimate of Fe requirement. Most of the Fe body pool is in the form of hemoglobin in red blood cells. At later stages of growth and maturity, the Fe requirement is likely to change as the blood volume declines and a lower rate of iron deposition occurs in tissues [1].

#### 2.2.2. Deficiency and Toxicity

Generally Fe deficiency causes anemia and tissue Fe depletion in fish and other vertebrates [30]. A characteristic microcytic anemia has been detected in brook trout [106,107], rainbow trout [107], Atlantic salmon [51,108], red sea bream [109,110], yellowtail [110], eels [63] and carp [66] fed low-iron diets. In most cases, semi-purified diet without Fe supplementation did not affect the growth of fish, except cobia, where fish fed a basal diet without Fe supplement (45.8 mg kg^−1^) showed a decrease in weight gain and feed efficiency [72]. In addition, a decrease in Hb and serum catalase activity was also observed. In catfish, Fe deficiency suppressed hematocrit, hemoglobin and plasma iron levels and caused transferrin saturation [55].

Effects of Fe toxicity in fish and animals have been comprehensively reviewed [102] and are briefly mentioned in this section. Dietary Fe toxicity was experimentally produced in rainbow trout fed levels higher than 1380 mg Fe kg^−1^ [107], a concentration far above the level found in ingredients used to formulate either practical or purified diets. The major effects of Fe toxicity were reduced growth, poor feed utilization, feed refusal, increased mortality, diarrhea and histopathological damage to liver cells. Iron toxicity occurs from excessive Fe exposure of fish in water, which interferes with Fe homeostatic regulation by causing Fe overload in tissues [102]. Iron naturally exists as soluble ferrous (Fe^2+^) and insoluble ferric particulate iron (Fe^3+^). In oxygenated waters, soluble Fe^2+^ oxidizes to Fe^3+^ and in circumneutral waters (pH > 6.5), Fe^3+^ ions are insoluble and rapidly precipitate as hydroxides and oxyhydroxides. Iron toxicity in water is closely related to Fe speciation and the interaction of Fe with body and gill surfaces. Excess Fe in the water is known to cause respiratory disruption due to physical clogging of the gills [111].

#### 2.2.3. Bioavailability

Iron in feeds is in two forms, heme iron and non-heme iron. Feed ingredients of animal origin (e.g., fish meal, animal meat and blood meal) are the major sources of heme iron. Non-heme iron refers to other sources of iron within feeds or inorganic contaminants from ingredient and/or feed processing. In cereal grains, a small proportion of Fe may be present as an iron phytin complex. Several factors are known to affect Fe absorption, including the amount and chemical form of Fe, Fe status and age of the animal, physiological conditions of the gastrointestinal tract (e.g., pH) and other dietary components (e.g., phytic acid, ascorbic acid, citrate) [112]. In juvenile animals, growth, hemoglobin concentration, plasma Fe and its retention may respond linearly to Fe supplementation; however, Fe repletion in deficient animals is considered the preferred method to estimate iron bioavailability [13].

Little is known about the bioavailability of Fe from feed ingredients and inorganic/organic iron feed supplements for fish. Bioavailabilities of ferrous sulfate and ferric chloride are considered to be essentially the same [6]. In red sea bream, ferrous and ferric chloride were more efficiently utilized than ferric citrate [113]. The biological availability of Fe measured by a hemoglobin regeneration assay in Atlantic salmon showed that the relative availability of Fe from ferric chloride, ferric oxide, blood meal and herring meal was 98.8, 17.8, 52.3 and 47.1%, respectively [52]. However, higher bioavailabilities of iron from blood meal [98], Fe-hydroxy methionine analogue [114] and Fe-methionine [72] have been reported for Atlantic salmon, grouper and cobia, respectively. The quality of blood meal varies widely in terms of protein quality. Whether the wide range of temperature and processing conditions used to produce blood meal affects the bioavailability of Fe remains to be investigated. There is also a need to standardize the method to predict the bioavailability of Fe in fish diets.

### 2.3. Manganese

Manganese plays an important role in protein and energy metabolism, bone mineralization, glycosaminoglycan synthesis, cellular defense against free radicals and metabolic regulation [115]. The essentiality of Mn in the above biochemical processes is based on its function as an enzyme activator (e.g., oxidoreductases, lyases, ligases, hydrolases, kinases, decarboxylases) and constituent of several metalloenzymes [116]. Many enzymes activated by Mn can be also activated by other metals, particularly Mg, with the exception of glutamine synthetase, glycosyltransferases, farnesyl pyrophosphate synthetase and phosphoenolpyruvate carboxykinase, which show specific Mn activation. Manganese metalloenzymes include arginase, pyruvate carboxylase and Mn superoxide dismutase (MnSOD). Limited information exists on the physiological aspects of Mn uptake from both gills and intestine and its metabolism in fish. In mammals, gastrointestinal absorption and biliary elimination of Mn are the two main regulatory sites of Mn homeostasis, which are influenced by the dietary Mn intake [115,117]. Excretion of Mn in bile and feces has also been observed in Atlantic salmon [118]. Manganese and Fe compete for absorption sites. A small fraction of biliary Mn excreted into the intestine may be reabsorbed. Manganese is efficiently absorbed from the diet but the absorption may be reduced by high levels of Ca and P, fiber and phytate [6]. Divalent Mn entering the circulation system is removed rapidly by the liver. Reduced intestinal absorption, enhanced liver metabolism and increased biliary excretion are considered adaptive mechanisms during high dietary intake of Mn [115].

Manganese uptake from FW has been demonstrated from the gills and gastrointestinal tract [119,120]. Unlike other trace metals, the mechanisms of Mn uptake from the gills, gut, skin and other tissues and toxicity are poorly understood. Brown trout exposed to low concentrations of Mn readily accumulated this metal in the blood and other tissues (gills, epidermal mucus, liver, kidney, viscera, skeleton and brain) [120]. The Mn toxicity was affected by water hardness and low pH and a higher risk of Mn toxicity to fish has been observed in acidic and Ca-deficient water [120,121]. These conditions can markedly enhance the uptake and toxicity of Mn and other metals. High concentrations of Mn caused Na imbalance, reduced the absorption of Ca and P, affected carbohydrate metabolism and impaired the immune functions of fish [122,123]. They also caused oxidative stress, tissue damage, inflammation, neurodegeneration and disruption of homeostasis of other metals in fish [120,124].

#### 2.3.1. Requirement

Manganese requirements of fish range from 2.5 to 25 mg kg^−1^ diet (Table 2). Some of these differences are likely due to differences in Mn uptake in water and bioavailability of Mn in experimental diets. Different requirement values were found using three different forms of Mn additives for cobia: manganese sulfate, 15.4; Mn-glycine, 11.2; Mn-2-hydroxy-4-(methylthio) butyrate, 10.5 mg Mn kg^−1^ [125]. In addition to growth, several studies have shown that body and vertebral Zn provides a good estimate of Mn requirements. A meta-analysis of Mn requirements for several fish species estimated 10.7, 13.4 and 18.4 mg Mn kg^−1^ for weight gain, whole body Mn and vertebral Mn content, respectively [105]. It appears that broodstock fish require larger amounts of Mn than juvenile fish [126]. Other dietary (e.g., Ca, P, phytate) and physiological factors, particularly changes occurring in bone mineralization at various stages of development, should also be taken into account for the estimation of Mn requirements of fish [6].

#### 2.3.2. Deficiency

The deficiency signs of Mn have been experimentally produced in several fish species. In addition to reduced growth, Mn deficiency causes skeletal abnormalities in rainbow trout, carp and tilapia [53,135,136]. In studies designed to determine Mn requirements of certain fish species (Table 2), a low intake of Mn caused reduced body and/or vertebral Mn concentration, a sign of poor bone mineralization. Although Mn deficiencies have been shown to cause a decrease in the activity of several enzymes in mammals [115], to date, decreases in the activity of liver Mn-SOD activity have been reported in Atlantic salmon [128], tilapia [132], cobia [125], gibel carp [131] and yellow catfish [130]. Low levels of dietary Mn (2.4 mg kg^−1^) did not affect Mn-SOD activity in catfish [129]. A decrease in cardiac muscle Cu-Zn-SOD was also observed in rainbow trout fed Mn-deficient diet [137]. Mn deficiency in broodstock rainbow trout diets affected reproductive performance and caused poor hatchability of eggs [126]. Manganese is considered to be one of the least toxic of the essential trace elements. However, high concentrations of dietary Mn supplementation (1 g kg^−1^) caused changes in feeding behavior, a decrease in body Fe concentration and elevation in Zn concentration in the body and vertebrae of grouper [123]. Freshwater-borne Mn toxicity in brown trout caused histological changes in their olfactory nerve and brain [120]. The effects of dietary Mn toxicity on the olfactory system and brain function of humans and animals are well documented [138].

#### 2.3.3. Bioavailability

Dietary Mn absorbed from the gastrointestinal tract is generally low in monogastric animals [13]. Several manganese compounds are available for use in animal/fish feeds: manganous chloride (MnCl_2_.4H_2_O), manganous oxide (MnO), manganous sulfate (MnSO_4_.4H_2_O or MnSO_4_.H_2_O), manganese carbonate (MnCO_3_), manganese acetate, manganous hydrogen phosphate (MnHPO_4_.3H_2_O), manganese amino acid complex, manganese methionine complex, manganese amino acid chelate and manganese proteinate. However, the availability of only a few compounds has been tested and their availability may differ in various inorganic Mn salt supplements. Manganese in manganous oxide is poorly utilized by rainbow trout and Atlantic salmon [6,139]. The most commonly used source of Mn in fish feeds is manganous sulfate, monohydrate. The availability of Mn is low in manganous carbonate for carp [140]. Mn-glycine was effectively utilized by cobia, turbot and Atlantic salmon reared in SW [118,125,141]. Mn-glycine was better utilized by cobia and turbot than manganous sulfate [125,141]. The availabilities of Mn-methionine and Mn-2-hydroxy-4-(methylthio) butyrate were relatively high for turbot [141] and cobia [125], respectively. Phytic acid, like other divalent ions (Zn^2+^, Cu^2+^ and Fe^2+^), binds inorganic Mn and reduces its bioavailability [142]. Recently, Antony Jesu Prabhu et al. [118,143] found that dietary supplementation of 15 mg kg^−1^ of Mn-Gly lowered the digestibility of Zn and Cu. The environmental impact of undigested Mn excretion in natural water is widely recognized so the upper limit has been set for Mn in complete feeds in certain countries. In Europe, the maximum limit for fish feed is 100 mg kg^−1^ [144].

### 2.4. Selenium

Selenium as an essential micronutrient for salmonids as well as a toxicant in diets and water is widely recognized [145,146,147]. The essentiality of Se for several farmed fish species grown in FW and SW is now widely recognized [3,6,105]. In nature, inorganic Se is present in four different oxidation states: selenate, selenite, elemental Se and selenide. In all biological systems, these forms are converted into more bioavailable organic forms, mainly as the two seleno-amino acids selenocysteine (SeC) and selenomethionine (SeMet). Selenoproteins are responsible for diverse biological functions and they all contain at least one SeC [148,149]. A comprehensive study of the identification and comparative analysis of vertebrate selenoproteomes has shown more than 45 selenoproteins in mammals and among bony fishes as well as 38 selenoproteins in zebrafish [150].

SeC is present in vertebrates at the active sites of glutathione peroxidases, thioredoxin reductases, iodothyronine deiodinases and selenophosphate synthetases, and is an essential component of other selenoproteins, [148,151]. The biochemical functions of many of these selenoproteins are poorly understood in fish. In several organisms, there are eight glutathione peroxidases (GPXs), five of them are selenocysteine enzymes (GPX1, GPX2, GPX3, GPX4 and GPX6), whereas the other three (GPX5, GPX7 and GPX8) have a cysteine at their catalytic site [148,152]. The three best characterized groups of selenoproteins in fish include glutathione peroxidases, thioredoxin reductases and iodothyronine deiodinases. The GPXs are involved in hydrogen peroxide (H_2_O_2_) signaling, detoxification of hydroperoxides and maintaining cellular redox homeostasis. GPX1 is the most abundant selenoprotein, which is considered a potent antioxidant in the cell scavenging of toxic H_2_O_2_. This protection of cells from oxidative damage by degrading toxic H_2_O_2_ has been closely linked to health and disease prevention in animals, humans and fish [149,153,154,155,156,157]. Thioredoxin reductases are also important antioxidant enzymes that maintain cellular redox status. Selenium as an integral part of GPx and thioredoxin reductase interacts with certain micronutrients (e.g., a-tocopherol) that affect redox status (i.e., pro-oxidant and antioxidant balance). The third group of selenoproteins are iodothyronine deiodinases, which activate the prohormone thyroxine (T4) to the active thyroid hormone triiodothyronine (T3), and catalyze the inactivation of T4 to reverse T3 and T3 to diiodothyronine (T2) [148].

Fish absorb limited amounts of Se from the environment via the gills and skin under certain conditions; however, the gastrointestinal tract is the primary site for Se absorption [158,159]. Low concentrations of selenium are found extensively in aquatic ecosystems [159]. The uptake of Se as selenite across the gills is efficient at low waterborne concentrations [147]. Selenocysteine and selenomethionine are likely to be absorbed by an active amino acid transport mechanism, whereas selenite is absorbed by simple diffusion and selenate by a sodium-mediated carrier shared with sulfate [160]. Absorbed Se is associated with proteins in the plasma and transported to tissues. After absorption from the diet, Se is transported to the liver and metabolized to selenide and incorporated into selenocysteine for selenoprotein synthesis or converted to selenosugars or methylated metabolites for excretion. Fish and other vertebrates excrete Se via feces, but urine is the primary excretion route and likely plays a quantitatively important role in Se homeostasis [158,160].

Beneficial effects and toxicity of dietary Se supplementation in fish and animals are well documented [1,47,161,162]. The effects of dietary Se also show an increase in expression of selenoprotein P in rainbow trout and zebrafish [163,164,165,166]. In Se-adequate animals, the kidney and liver have the highest Se content. Muscle has moderate levels of Se content but accounts for the largest pool of body Se. Retention and distribution of Se in tissues are affected by the body Se status and chemical form of Se [167]. Selenium-deficient animals retain Se more efficiently than Se-adequate animals.

#### 2.4.1. Requirement

Selenium requirements of several fish species have been determined on the basis of different response criteria (growth, liver and plasma/serum GPx activities and Se concentration of total body and tissues, e.g., liver, muscle) and are summarized in Table 3. The lack of standard methodology and form of Se used makes it difficult to compare the results of different species or within species. The minimum Se requirement of fish varies with the form of Se (inorganic or organic) ingested, Se availability from different feed ingredients from the diet, vitamin E content of the diet and concentrations of waterborne selenium. The estimated Se requirements based on weight gain, whole body Se retention and liver GSH-Px activities have shown different values for some fish species. Some studies have used a single or limited number (<3) to test the requirements, which has limited value in assessing the Se requirement properly. Published data on the Se requirement of farmed animals have demonstrated that growth alone does not reflect their Se requirement. A meta-analysis of published information on Se requirements of several fish species showed different estimates for the Se requirement (mg kg^−1^) based on different parameters: weight gain, 0.35; enzyme activities of liver GPx, 0.78; serum GPx, 0.43; liver glutathione reductase (GR) activity, 0.41 [105]. The estimated requirement of Atlantic salmon smolts based on available Se in a plant ingredient based-diet was much lower (0.27 mg kg^−1^). In coho salmon, based on growth, whole body and liver Se contents, the dietary Se requirement was found to be in the range of 0.39–0.43 mg kg^−1^ [168]. Supplementation of Se-Meth as compared to sodium selenite showed lower Se requirements for Atlantic salmon smolts [169] and gibel carp [65]. In addition to differences between the bioavailability of Se from the experimental diets, Se uptake from water, age, dietary vitamin E levels and Se bioavailability and differences among fish species in the utilization of Se must be considered when published requirement values are applied in feed formulation.

#### 2.4.2. Deficiency

Unlike other micronutrients, gross deficiency of Se alone has not been characterized due to its interaction with vitamin E, polyunsaturated fatty acids and other dietary factors. Early clinical signs of deficiency caused by low dietary intake of Se have been detected in low enzyme activities in plasma and liver glutathione peroxidase of several fish species [145,146,170,171,172,173,174,175,176,177,180,181]. Selenium deficiency led to reduced growth in rainbow trout [146], carp [182] and catfish [170], but Se deprivation did not lead to any pathological signs in these species. A combination of dietary vitamin E and Se was found to prevent muscular dystrophy in Atlantic salmon [145] and exudative diathesis in rainbow trout [181]. Extensive research conducted on animals shows that it is very difficult to produce or distinguish symptoms of Se deficiency alone from that of a Se-vitamin E deficiency [1]. 

Although the effects of selenium deficiency on the reproduction of animals are well documented [1,162] the effects of Se on the reproductive performance of fish are not clear. Recently, Wischhusen et al. [183] found that Se supplementation of diets based on a major proportion of plant ingredients enhanced the total number of spawning fish and higher levels of hydroxy-methionine supplementation led to earlier spawning in rainbow trout. There was no evidence of transfer of maternal Se to progeny and supplementation of organic Se increased GPx activity and mRNA expression of other proteins involved in antioxidant protection at the cellular levels as well as elevated tissue vitamin C and E concentrations. 

#### 2.4.3. Toxicity 

Dietary Se toxicity has been extensively studied in farm animals and the following three possible mechanisms of toxicity have been proposed: (a) substitution of Se for sulfur in important biochemical reactions and structures (e.g., disulfide bonds may disrupt normal function and cell integrity); (b) reaction between selenite and glutathione depletes cellular free and protein-bound thiol levels, thus affecting the activities of certain enzymes; (c) free radicals such as superoxide anions produced by the reactions of certain forms of Se with tissue thiols may cause oxidative injuries to tissues [1,94]. In fish, both dietary and waterborne Se toxicities have been experimentally produced [147]. The physiological mechanisms involved in the uptake of Se from water and its toxicity to fish and other aquatic organisms have been extensively investigated and are the subject of comprehensive reviews [158,159,184]. Waterborne inorganic forms of Se (selenate and selenite) are not absorbed appreciably through gill membranes [185] and ingestion of natural food organisms that accumulate Se from the aquatic environment appear to be more toxic [159]. The susceptibility to Se toxicity and the mode of action of Se toxicity may differ among fish due to their diverse chemical properties, uptake and metabolism from diet and water. Coho salmon are more sensitive than chinook salmon to inorganic Se [186]. As in the case of trace element bioavailability, the relative toxicity of a given Se compound is affected by its chemical form and solubility. Highly insoluble elemental Se is much less toxic to many species than other more soluble forms such as selenite and selenate [47,158]. Dietary selenomethionine (Se-Meth) was less toxic to chinook salmon, coho salmon and Atlantic salmon than selenite or selenate [186,187]. However, the Se-Meth caused Se toxicity in white sturgeon fed levels above 20.5 mg Se kg^−1^ diet [188].

A relatively narrow range between dietary requirement and toxicity exists in fish and other vertebrates [146,158,162]. Selenium toxicity has been reported in rainbow trout and catfish when the dietary Se level exceeds 13 and 15 mg kg^−1^ dry feed, respectively [146,170]. Reduced growth, poor feed efficiency and an increase in mortality have been reported as the major adverse effects of Se when concentration exceeded the dietary requirements established for juvenile fish. In addition to reduced growth, several other adverse effects of feeding higher levels of selenite and Se-Meth in diets include decreased energy retention [189,190], lower egg viability [191], decrease in swimming activity [188], reduced immunological functions [155], pathological changes in liver, kidney and ovaries [184,192] and skeletal deformities [167,187,193].

For the assessment of early sublethal adverse effects of selenite and Se-Meth toxicity, several biomarkers such as tissue lipid peroxidation, reduced glutathione (oxidative stress marker) and changes in lipid composition have also been proposed [167]. Elevated Se levels for rainbow trout, chinook salmon, fathead minnow, striped bass, bluegill and razorback sucker ranged from 2.4 to 70 mg Se kg^−1^ in feed and 47 to 472 ug L^−1^ of water [184]. In most cases, reduced growth or survival occurred at dietary Se levels close to 3 mg Se kg^−1^. Atlantic salmon tolerated either 1–2 or 3 mg Se kg^−1^ of selenite and Se-Meth supplementation, respectively, in a feed based on a high proportion of plant ingredients that contained 0.45mg Se kg^−1^ [187]. The relative toxicity of different Se supplements may be largely related to their solubility in water and nutrient bioavailability and can be modulated by dietary factors such as protein, sulfate, vitamin E and a number of trace elements including As, Cu and Hg. The maximum limit for total Se in animal feeds, including fish, has been set at 0.5 mg Se kg^−1^ feed by the U.S. Food and Drug Administration and the European Union [194], which is considered low to meet the Se requirement of Atlantic salmon fed diets based on plant ingredients and low in fish meal [169].

The primary biochemical mechanism for chronic Se toxicity was initially considered to be linked to the substitution of Se for sulfur in cysteine and methionine, which affected the tertiary structure of protein and its function by altering disulfide linkages [195]. Oxidative stress has been proposed as a main cause of excess dietary Se intake or exposures in fish [167]. In Atlantic salmon fed high levels of selenite and Se-Meth yeast, oxidative stress was a main driver for Se toxicity [167]; however, white sturgeon and brown trout fed high levels of organic Se did not show oxidative stress [190,196]. Altered liver lipid synthesis and metabolism have been shown to be the central mechanism in dietary organic Se toxicity in rainbow trout [196,197]. Wide-scope pathway assessments by the application of metabolomics techniques show that disturbance in lipid metabolism is an important factor in inorganic and organic Se toxicity [167].

#### 2.4.4. Bioavailability

Feed sources of Se used in feeds are either in inorganic (selenite, SeO_3_^2−^, or selenate, SeO_4_^2−^) or organic (selenized yeast, Se-Meth and analogues) forms and they differ significantly in bioavailability, metabolism and toxicity. The major proportion of Se in common feed ingredients occurs as seleno-amino acids with Se-Meth being the most predominant form [1]. In addition, other minor organic forms derived from plants or yeast metabolism such as Se-adenosylselenohomocysteine, methylselenocysteine, selenocystathionine and γ-glutaminyl-Se-methylselenocysteine have been reported [198,199]. The Se content of feed ingredients of plant origin varies in various geographical locations to a great extent, depending on the Se concentration of the soil and its uptake. In cereal grains, it may range from <0.1 to >0.8 mg kg^−1^ [3,200]. Fish meals represent the best natural source of Se among the common feedstuffs with concentrations ranging from 1–2.4 mg kg^−1^, with the exception of tuna and mackerel meals where concentrations may exceed 5 mg kg^−1^ [3,200]. High concentrations of Se have also been reported in shrimp meal and crab meal [3,200]. Selenium is widely distributed in small concentrations in FW (0.1–0.3 ug L^−1^) and SW (0.05–0.2 ug L^−1^). In certain regions of the USA with highly seleniferous exposed shale deposits, levels as high as 5–50 µg Se L^−1^ in water have been reported [201]. Limited amounts of dissolved inorganic forms of Se as selenate and selenite in water are absorbed through gill membranes [185].

Several factors affect the bioavailability of Se, including form of Se, other dietary components, physiological status of Se in animals and species differences [202,203]. The effects of the chemical form of Se in feeds and feed ingredients on the bioavailability and metabolism of this element have been extensively studied in animals [202] and humans [204]. Different criteria have been used to determine the bioavailability of Se including GSH-Px activity, tissue Se concentration and prevention of Se deficiency. The activity of GSH-Px in plasma, red blood cells and a number of tissues responds to dietary Se concentration. Organic sources of Se supplements have shown a higher bioavailability as compared to inorganic forms for fish [163,169,171,205,206,207,208]. Selenium-enriched yeast showed higher bioavailability than sodium selenite [209]. Nanoforms of Se also show high bioavailability in vertebrates [162]; however, their bioavailability, metabolism and safety remain to be fully evaluated in fish [156].

The early work of Bell and Cowey [210] showed higher bioavailability in Atlantic salmon and it follows the order from highest to lowest: selenomethionine > selenite >selenocysteine > fish meal. Certain fish meals, e.g., tuna, may have poor biological availability because of heavy metal complexing of Se. A wide variation in Se availability (38.5–60%) of different batches of capelin meal measured using GSH-Px activity in a chick bioassay was observed [211]. Until the initiatives to reduce the amount of fish meal with plant ingredients, Se supplementation of farmed fish diets was not considered necessary. An increase in plant protein sources and reduction in fish meal in feeds have reduced the level of Se and bioavailability of Se [163,169,206,209,212,213,214]. Selenium supplementation of fish diets appears to be the most effective method to meet its requirement.

### 2.5. Zinc

Zinc is the second most abundant trace element, after Fe, essential to all cells in most living organisms [215]. It has many diverse biochemical functions and is the subject of extensive studies to define the role of Zn at the subcellular level in nutrition and health of human and animals. Most knowledge related to the biochemical function of Zn has emerged from research on other vertebrates and this area is wide open for research from fish perspectives. The ubiquitous distribution of Zn among cells, coupled with it being the most abundant intracellular trace element, has resulted in identification of three specific functions in biology of vertebrates and plants: (a) catalytic, (b) structural and (c) regulatory. The catalytic role of Zn is essential for the biological function of all six classes of more than 300 enzymes [216]. Some examples are RNA nucleotide transferases (RNA polymerase I, II and III), alkaline phosphatase and carbonic anhydrases. In the structural role of metalloenzyme, Zn ion stabilizes the tertiary structure of enzymes (e.g., Cu-Zn superoxide dismutase). For this enzyme, Cu serves at the catalytic site and Zn serves a role in structure. Zn finger (where some histidine replaces cysteine) motifs in protein represent an important structural role. The single Zn atom at the base of the motif influences the binding of protein to DNA. The linking of these Zn fingers to the corresponding sites on DNA initiates the transcription factor and initiates gene expression. Approximately 3000 Zn proteins in the human genome and fish genome carry “Zn binding” annotation [217]. Zinc is required for the structural and functional integrity of over 2000 transcription factors and almost every signaling and metabolic pathway is dependent on one or more zinc-requiring proteins [218]. Additionally, Zn as an intracellular regulatory ion activates or inhibits transcription factors responsible for regulating gene expression. An example of this role is metallothionein (MT) or MT-like proteins.

The homeostasis of Zn is controlled at the whole body, tissue, cellular and subcellular levels by different proteins involving zinc transporters. Two Zn transporter families, Zn transporters (ZnT) and Zrt/Irt-like proteins (ZIP), function in the mobilization, influx, efflux, compartmentalization and sequestration across biological membranes [219]. ZnT and ZIP thus contribute to a wide range of physiological and cellular functions (e.g., immune, endocrine, reproductive, skeletal and neuronal) by tightly controlling zinc homeostasis [219,220]. Biochemical roles for Zn transporters and regulation of specific genes have mainly been studied in the zebrafish model [221,222].

Like other trace elements, the main routes of zinc uptake from water are through the gills and gastrointestinal tract; however, the major route of Zn absorption is the gastrointestinal tract both in FW and SW [223]. Waterborne and dietary Zn is also a potential source of this mineral for salmonids and marine fish because they drink SW [224,225]. In FW fish, uptake across the gill can contribute significantly (~50%) to total Zn absorption if the Zn concentration in the water is high or that in the diet too low. Zn may interfere with calcium homeostasis by competitive inhibition of Ca^2+^ transfer across the apical membrane of the gill epithelial cells by Zn^2+^ [226]. An excessive uptake and accumulation of Zn in the gills is also regulated through alteration in Zn uptake mechanisms [14]. Chelation of Zn with amino acids, such as histidine or cysteine which have a high affinity for this element, may enhance Zn absorption and distribution in the tissues of fish [97]. The excretion of dietary Zn through gills has also been observed in rainbow trout [227].

Waterborne Zn^2+^ shares part of a common pathway with Ca^2+^ for uptake in the gills of FW fish [19] and Ca may have either inhibitory or stimulatory effects on Zn absorption depending on concentration [83,217]. An interaction between high levels of dietary Ca and Zn in water may protect FW fish from Zn toxicity [228]. Zinc accumulation in gills also affects additional Zn uptake from water and is considered an important physiological mechanism to maintain Zn homeostasis by limiting its excessive absorption [14]. In addition, the control of absorption by the gastrointestinal tract and excretory mechanisms plays an important role in Zn homeostasis. Zinc is excreted via bile in urine, the sloughing off of intestinal mucosa in feces and gills in fish [19]. Limited information exists on absorption of dietary Zn from the gastrointestinal tract and transport mechanisms as free Zn ions or bound to certain amino acids [14,18]. Information on the absorption of Zn from the gut is limited. In the intestine, dietary Zn binds to the mucus of the intestinal epithelium, and is transported into the epithelial cells either as Zn ions or as ions bound to amino acids [14,18]. It is well known that high intakes of phytate and iron reduce the absorption of zinc [6]. In vitro studies using a recently developed enterocyte model have the potential to better understand the mechanisms involved in the absorption of inorganic and organic Zn in fish feeds and Zn supplements [229].

#### 2.5.1. Requirement

A dietary requirement for Zn has been reported for several juvenile fish species (Table 4). The minimum Zn requirement varies with age, sexual maturity, composition of diet, water temperature and water quality [3]. The response criteria used to determine Zn requirements of fish in various studies include growth, feed efficiency, deficiency signs, whole body Zn concentration and retention, serum or plasma levels and enzyme activities. Most of the above criteria show responses to increasing dietary concentration, and the major proportion of dietary Zn is retained in skeletal tissues, particularly vertebrae. A meta-analysis of published information on Zn requirements of several fish species showed estimates ranging from 33.5–64.6 mg kg^−1^ on the basis of different parameters tested (weight gain, 36; whole body Zn, 33.5; vertebrae Zn, 64.6; serum Zn, 53.4; serum ALP activity, 47) [105].

#### 2.5.2. Deficiency

Overt signs of Zn deficiency are difficult to produce in short-term experiments due to the ubiquity of this trace element in water and feed ingredients. Early work of Ogino and Yang [231] showed lens cataracts, erosion of fins and skin as well as growth depression and high mortality in juvenile rainbow trout fed semi-purified diet containing a low level of Zn. Satoh et al. [136,182] observed short body dwarfism due to poor mineralization of trout vertebrae. In several fish species, low dietary Zn intake caused reduced growth, low serum, liver, scale, body and vertebral Zn concentrations. Widespread occurrences of cataracts in salmonids fed diets based on high amounts of white fish meal in United States hatcheries were attributed to Zn deficiency [249]. Zinc is considered essential for normal eye development in juvenile fish and high levels of dietary Ca and P in fish meal and phytic acid in plant ingredients reduce Zn bioavailability, resulting in lens cataracts [232,249,250]. Dietary histidine has also been found to prevent development of cataracts in Atlantic salmon smolts [251]. Zinc and multiple dietary, genetic and environmental factors may be involved in the pathogenesis of cataracts in Atlantic salmon [30,252,253]. Broodstock diets low in Zn reduced egg production and hatchability of eggs in rainbow trout [126]. Caudal fin Zn concentration is considered a good indicator of Zn status in rainbow trout [254].

#### 2.5.3. Toxicity

Excessive dietary zinc levels may become toxic to fish and compete for similar binding sites with other bivalent minerals such as Cu, Fe, Ca and Cd in the digestive tract during absorption [80,234]. Rainbow trout and carp can tolerate 1700 to 1900 mg zinc kg^−1^ in the diet without any apparent signs of toxicity [255]. Common carp accumulate higher concentrations of Zn in their tissues, particularly in the viscera, than other fish studied, without any overt toxicity signs [256]. In rainbow trout, high concentrations of dietary Zn (500 to 1,000 mg Zn kg^−1^) caused reduced hemoglobin, hematocrit and hepatic Cu concentrations in rainbow trout [49].

Effects of environmental pollution and heavy metal contamination of aquatic organisms have been the subject of intensive research, particularly their accumulation in fish [257]. Uptake and toxicity of zinc and other metals from water are known to vary, depending on the physiological status and osmoregulatory mechanisms [232,258]. The toxic effects of Zn have been studied mainly in FW, which show that hypocalcemia caused by Zn interferes with active Zn uptake at the gills [18]. Sublethal Zn exposure of killifish in FW and SW caused pathological changes in both Ca and Na homeostasis and an increase in salinity exerted protective effects against sublethal and lethal Zn toxicities [258,259]. Mineral sensitivity is highest during the larval stage, compared to other times in the life history of a fish. The zinc content of scales reflects environmental metal concentrations [18]. 

#### 2.5.4. Bioavailability

Dietary factors (e.g., form of Zn, protein source, phytic acid, and dietary Ca and P levels) are known to affect absorption and retention of Zn in fish [214,227,232,233,241,250,254,260,261,262]. In most Zn requirement studies (Table 4), zinc sulfate (ZnSO_4_·7H_2_O) has been used due its high bioavailability. Both zinc sulfate and zinc nitrate (40 mg kg^−1^) alleviated dwarfism and cataract problems in rainbow trout [260]. The bioavailability of Zn oxide is low in Atlantic salmon and other fish species, probably due to the lower solubility of this compound [6]. Recent studies on monogastric animals show high variability of zinc oxide feed supplements in color, texture and Zn content and manufacturing techniques, which affects their bioavailability [263]. Organically complexed minerals (amino acid chelate, yeast complexes, etc.), including zinc, appear to be more readily available to rainbow trout compared with inorganic sources [205,262,264,265,266]. No apparent differences in the bioavailability of zinc sulfate and zinc methionine were observed in catfish [267]. In gilthead seabream, zinc oxide was found to be very effective for eliciting good whole body growth response, whereas the chelated form appeared to induce greater antioxidant responses [268]

Although fish meal produced from whole fish (e.g., herring and capelin meal) is considered a good source of Zn (80 to 130 mg Zn kg^−1^) and other minerals, the concentration of Zn varies in meals produced from processing discards containing partial fish parts. Feed ingredients of animal origin (meat and bone meal, poultry feather meal) contain high zinc levels (90^−1^40 mg Zn kg^−1^). High levels of Ca and P in high-ash fish meal and meat and bone meal affect the bioavailability of Zn [6,11,139,269]. Phytate in plant products, especially cereals and legumes, irreversibly binds zinc in the intestinal lumen and reduces its bioavailability. Furthermore, there are copper–zinc and calcium–phytate–zinc antagonistic interactions, which can decrease bioavailability of Zn to rainbow trout [6,11,139,269,270]. Fiber may interfere with Zn absorption but this may be attributed to the phytate content of high-fiber plant feedstuffs. Amino acids, such as histidine and methionine, and other low-molecular-weight ions, such as EDTA and organic acids (e.g., citrate), are known to have a positive effect on Zn absorption [270]. The removal or reduction of phytate by enzyme (phytase) treatment, fermentation or plant breeding/genetic engineering markedly improves Zn absorption [271]. Increasing levels of Cd in foods associated with environmental contaminants and other factors has also been shown to reduce zinc absorption [270]. A higher limit in fish feed has been set for salmonids (150 mg kg^−1^ of diet) and other fish (100 mg kg^−1^ of diet) in Europe [263].

### 2.6. Iodine

Iodine is an essential constituent of the thyroid hormones T3 (3,5,3′-triiodo-L-thyronine) and T4 (L-thyroxine; 3,5,3′,5′-tetraiodo-L-thyronine) that regulate cell activity and growth in all tissues. The metabolism of thyroid hormones (THs) and iodine which mainly exists as inorganic iodide are closely linked, and THs play a critical role in cellular oxidation, hematopoiesis, circulation, reproduction, neuromuscular functioning and metabolism of major nutrients [272]. T3 is the predominant hormone secreted by the thyroid gland and is regarded as an active precursor for T4. T3 is more biologically active than T4 in several fish species [273,274]. The inhibition of extrathyroidal T4-to-T3 conversion reduces the potency of thyroid hormone [275,276]; however, the mode of action of the less active T4 is not fully established. Thyroid has an established role in development and metamorphosis [277,278], and where TH metabolism could differ. Major differences exist between fish and mammals in the physiology of I and extrathyroidal metabolism of T4 and T3 as reviewed by Eales [279].

The branchial uptake of iodide by fish is widely recognized. Marine fish drink SW, ensuring an adequate intestinal I absorption, which is supplemented by I in food and possible I uptake through body surfaces [280]. FW fish drink negligibly, hence, dietary and gill uptake are both considered important sources of I [273,281]. Most research on I metabolism in fish has been focused on salmonids in the FW phase of their lifecycle. In rainbow trout, approximately 19% I is derived from diet, 80% from water, and less than 1% from recycling iodide originating from thyroid hormone degradation [282]. Salmonids undergo parr–smolt transformation, with established thyroid involvement [283]. After the growth phase during sexual maturation and reproduction, some changes in thyroidal status occur as well as interaction of THs with sex-related hormones [274,284]. Female fish transfer significant amounts of thyroid hormones to developing ova [277,285] and the offspring may rely on parental I storage to complete their early development. Some of these physiological changes may affect the I metabolism and requirement at different stages of the life cycle of fish [273].

Iodine enrichment of live food organisms such rotifers, copepods and Artemia to increase their I concentration has been tested with cod, halibut and Senegal sole larvae [286,287,288]; however, its retention was low in some species, probably due to differences in the bioavailability of I from water or the form of I compounds used to increase the I concentration [289]. Penglase et al. [288] have, however, clearly demonstrated that rotifers could be successfully enriched with I. Feeding cod larvae a high concentration of I-enriched rotifers (129 mg I kg^−1^ DW) caused I toxicity. Ozone treatment of sea water in the recirculation systems oxidizes I to an unavailable IO^3−^ form, which has low bioavailability for fish [290]. Nitrate (NO^3−^) is considered goitrogenic for fish and as its build-up in water may block iodide uptake by the sodium iodide symporter [291], which can even cause goiter in sharks [292].

The relation of I deficiency to enlargement of the thyroid gland or goiter in salmonid fish was first shown by [293]. Senegalese sole larvae developed thyroid hyperplasia and hypertrophy when fed Artemia and grown in a recirculation system [287]. In vertebrates, other deficiency signs of I include goiter, cognitive and neuromuscular retardation, embryonal and postnatal mortality and impaired fertility [294]. Excessive I intake can also negatively affect thyroid hormone production and produce goiter, termed I or colloid goiter in humans [295]. In most fish species, I requirement and deficiency remain to be investigated. Woodall and LaRoche [296] found a higher iodine requirement for advanced parr compared to fingerlings due to increased thyroid activity during smoltification. Lall et al. [297] observed that 4.5 mg I kg^−1^ of diet was essential to protect Atlantic salmon from bacterial kidney disease infections. It is likely that I requirement is influenced by growth, sex, age, physiological status, environmental stress, disease and iodine content of the water.

Few definitive studies on iodine bioavailability have been conducted due to high uptake of I from water and the problem with distinguishing absorption from water and dietary sources. Iodine concentration of marine fishes is relatively high [298,299]; however, substantial amounts of iodine are lost during fish meal processing [6]. Ingested inorganic iodine and iodate are reduced to iodide and absorbed almost completely from the gastrointestinal tract [272]. Certain seaweeds also contain high levels of iodine [300]. Goitrogenic substances in feed may increase iodine requirements depending on the amount and type of this natural toxicant [301]. Glucosinolates (GLSs) in rapeseed meal are known to impair thyroid function, causing goiter in vertebrates [302] and possibly also in fish [273,303]. This effect is caused by their hydrolytic products (e.g., thiocyanate anions, visnyloxazolidinethiones and isothiocyanates). The thiocyanate anions are competitors of iodine for active transport across the cell membrane and for binding to tyrosine residues of thyroglobulin. Burel et al. [304] showed that dietary supplementation with T3 or iodine induced an increase in plasma T3 levels, as compared to fish fed rapeseed meal diets, and reduced the deleterious effect of rapeseed meal (RM) on growth. Processing methods as well as novel varieties have been developed to overcome these antinutritional factors in rapeseed products [305].

### 2.7. Chromium

Chromium is a transition metal that exists in food and the environment as Cr^3+^ (trivalent) and Cr^6+^ (hexavalent) forms. These naturally occurring oxidation states differ significantly in their bioavailability and toxicity. Trivalent Cr has been postulated to be involved in regulating carbohydrate and lipid metabolism by enhancing insulin’s efficacy [306]. No Cr-dependent enzymes have been identified. The precise biochemical mechanism of Cr as an essential trace element is not clearly known; however, it has been shown that Cr binds to an oligopeptide to form chromodulin, a low-molecular-weight, chromium-binding substance that binds to and activates the insulin receptor to promote insulin action [307]. It may also have antioxidant effects. Recent research has suggested that although pharmacologic amounts of Cr as a therapeutic agent might increase insulin sensitivity and affect lipid metabolism, it is not an essential mineral [307,308]. This is because, according to the definition of an “essential trace element”, its absence or deficiency from the diet does not produce abnormalities that can be reversed with the addition of Cr. Molecular mechanisms have been proposed for the beneficial effects of Cr but have not been definitively shown to occur consistently in all animals.

Chromic oxide is commonly used as an inert tracer in studies on measuring apparent digestibility coefficients with fish or shrimp. Some specific studies on the effect of Cr in fish have been related to its role in metabolism [309,310,311,312,313,314,315,316], growth [317,318] and toxicity [319]. To date, many of these studies have provided some evidence that Cr has an effect on the metabolism of fish; however, the Cr forms tested and their level as well as experimental conditions were different in these reports. Chromium yeast appears to modulate the immune response of rainbow trout, and this effect was both dose and time dependent [320]. Information on the need for supplemental chromium in practical diets of certain animals including fish was too sparse to allow any conclusions [47]. A need for research designed to create reproducible signs of chromium deficiency in animals, which would facilitate the establishment of dietary chromium requirements, was identified.

Chromium is absorbed across the gills and transported via blood to tissues but mechanisms of absorption from gills and the gastrointestinal tract and excretion are not known. Studies on the uptake of Cr from water as it relates to physiology and toxicology have been reviewed [321]. The toxic Cr^6+^ readily passes through cellular membranes and is then reduced to the trivalent form. This Cr^3+^ combines with several macromolecules including genetic material inside the cytosol, and ultimately exposes the toxic and mutagenic alterations of Cr toxicity. Higher levels of Cr in diet and water caused histological changes in the intestine, gills, liver and kidney but the mechanism of toxicity remains to be established [321,322].

### 2.8. Cobalt

Cobalt is a component of vitamin B_l2_, collectively called “cobalamins”. Methylcobalamin and 5-deoxyadenosylcobalamin are the metabolically active forms of this vitamin, however, two other forms, hydroxycobalamin and cyanocobalamin, are converted to the active forms methylcobalamin and 5-deoxyadenosylcobalamin. Microbiota in the digestive tract of ruminants and algae are known to synthesize vitamin B_l2_ from inorganic cobalt sources. Monogastric animals and fish require vitamin B_12_ because they lack the ability to synthesize this vitamin from dietary Co in sufficient amounts by microbiota in their digestive tract. In certain warmwater fish, intestinal synthesis of vitamin B_12_ by microorganisms appears to satisfy the requirements of this vitamin for Nile tilapia [323,324] and hybrid tilapia [324], but not for channel catfish [325]. The estimated dietary Co requirement reported for Tilapia zillii was about 100 mg Co kg^−1^ of diet [326]. A lower concentration of Co (10 mg Co kg^−1^) in the diet promoted gastrointestinal bacterial synthesis of vitamin B_12_ in Malabar grouper and met the dietary requirement of this vitamin [327].

Cobalt is absorbed by FW fish via the gills and gut as the main routes of uptake [328,329]. Some uptake of cobalt occurred in rainbow trout eggs during embryonic development [330]. The uptake routes, homeostasis and mechanism of Co toxicity have been reviewed [329]. The levels of Co normally present in common feed ingredients, animal and fish diets are relatively low and do not cause toxicity [1,47]. 

### 2.9. Boron

Boron is an essential nutrient for plants as well as algae, but biological functions required to establish its essentiality for humans and animals are not clearly identified. To date, boron has been found to be essential for only zebrafish to complete their life cycle [331]. It also stimulates embryonic growth in trout [332] and zebrafish [331]. Boron has beneficial effects on such functions as reproduction and development, calcium metabolism, bone formation, brain function, insulin and energy substrate metabolism, immunity and the function of vitamin D and steroid hormones [333,334]. As compared to FW, the concentration of boron in the marine environment is relatively high (0.4 mM), which is efficiently taken up by algae [335]. Acute toxicity of B to fry of Chinook salmon and Coho salmon has been reported [186]. 

### 2.10. Cadmium

Cadmium is a heavy metal that does not have a clear physiological function as a nutrient and is considered a toxicant for fish by its uptake from the aquatic environment. The toxicity of Cd causes the disruption of Ca ion homeostasis and to some extent Na and Mg [25]. Cadmium enters the gill epithelium via the same pathway as Ca^2+^ (apical Ca^2+^ channel of the chloride cells) and inhibits basolateral Ca^2+^ ATPase, thereby blocking active Ca^2+^ uptake [336]. Atlantic salmon fed 25 mg Cd kg^−1^ showed inhibition of ATP-dependent Ca uptake measured as Ca^2+^and Na^+^/K^+^-ATPase in the intestine [87]. Generally, the concentration of Cd in most feed ingredients is relatively low [337].

### 2.11. Arsenic

The biochemical role of arsenic as an essential micronutrient for fish and higher animals is not clearly established; however, its dietary deprivation affects the physiological functions of certain animals [47]. Arsenic is ubiquitous in nature as an oxyanion with an oxidation state of either 3^+^ or 5^+^, but it also forms compounds where As has an oxidation state of 3^-^. In general, it is trivalent. As compounds, inorganic (arsenite) and organic (monomethyl arsenic) forms are considered more toxic than pentavalent compounds. More than 25 different arsenic species have been identified in marine biota [338]. Arsenic binds covalently with most metals and non-metals and forms stable organic compounds. In fish and animal tissues, inorganic As occurs mainly as arsenate, and in the methylated form as dimethylarsinic acid and monomethylarsonic acid. Arsenic in algae is transferred via the food chain to other aquatic organisms [338]. The major organic arsenic species in fish and other marine organisms are arsenobetaine and arsenocholine [339], and in seaweed and microalgae, aresenosugars (dimethylarsinoyl ribosides) [340]. About 80% or more of As in fish is organic As, with arsenobetaine being the dominant species in marine fish [47]. In freshwater fish, As concentration is much more variable than in marine fish. Lipids of marine fish and other marine organisms contain As as arsenolipid and have been comprehensively reviewed [341]. 

Arsenic is far less toxic to fish and other vertebrates [47] than most other metals. The incorporation of As in the diet of rainbow trout to induce toxicity showed reduced growth and As accumulation in several tissues [342,343]. Arsenic concentration of feed ingredients of terrestrial and aquatic origin used in fish feeds depends on As uptake from soil by plants and aquatic food organisms consumed by fish, which varies widely in different regions of the world. Fish products including meal and oil show a wide range of As concentrations [344]. In a study designed to investigate the As content of fish meals and fish oils of two different geographic origins, Sissener et al. [212] found a wide variation in As concentration of North Atlantic (9.9 mg As kg^−1^) and South American (9.9 mg As kg^−1^) fish meals. However, the fish oils from these two origins showed relatively similar As levels (~9.3mg As kg^−1^). Insect meals produced from insects raised on seaweeds showed an increase in As concentration, which was detected in Atlantic salmon fed diets based on these meals [345]. Effects of As contamination of common food sources including seafood and water have been the subject of major concern from the perspective of human and animal health and are beyond the scope of this review.

### 2.12. Other Trace Elements

Biochemical functions of other trace elements (F, Li, Ni, Pb, Si and V) have been shown in animals and humans, but their dietary essentiality based on the defined criteria of physiological impairment has not been widely accepted. These minerals have been studied in fish mostly from physiological aspects of their uptake from water and toxicity. 

## 3. Macrominerals

### 3.1. Calcium and Phosphorus

Calcium and phosphorus play a major role in the development and maintenance of the skeletal system and perform many other physiological functions including the maintenance of acid–base equilibrium [346]. In skeletal tissue, Ca and P are deposited as tricalcium phosphate Ca_3_(PO_4_)_2_, which then undergoes further crystalline changes to form hydroxyapatite, Ca_10_(PO_4_)_6_(OH)_2_, which is deposited in the organic matrix during mineralization. The ratio of Ca to P in bone may show some changes during development, however, their ratio reported in several fish species ranges from 1.6:1 to 2:1. Fish scale is also a calcified tissue and serves as an internal Ca reservoir during periods of increased Ca demand, such as sexual maturation and starvation [347]. During the reproductive period, the plasma Ca level increases in females, which is bound to vitellogenin, a major component of egg protein and a calcium-binding protein [348].

Fish absorb Ca and P from the surrounding aquatic environment via gills, gastrointestinal tract and integument; however, the gills represent the major site of Ca uptake [7]. The physiological aspect of Ca uptake at the gills is well established and is the subject of several reviews [349,350,351]. The absorption and metabolism of Ca depends not only on its concentration in the surrounding water, but it is affected by species differences and their homeostasis by the endocrine system, biological availability from diet and P level [96]. Osmoregulation allows them to control their Ca levels predominantly via the hypocalcemic hormones stanniocalcin and calcitonin. Certain minerals (e.g., Cd, Cu, Mg, Sr, Zn) may reduce Ca absorption from gills or gastrointestinal tract. In vertebrates, vitamin D is known to play an essential role in Ca metabolism. Although limited research has been conducted on fish, the function of the endocrine system and metabolites identified appears similar in fish and terrestrial vertebrates [328,352,353,354]. 

In addition to skeletal tissue metabolism, P as phosphate (HPO_4_^2−^) plays a major role in the function of all cells. It is a major signaling molecule, and a structural component of cell walls, essential for the nucleic acid helical structure (i.e., RNA and DNA), and a component of high-energy compounds (i.e., AMP, ADP and ATP). Food is the main source of P for fish because FW and SW are low in phosphate. Thus, regulation of phosphate is considered more critical than that of Ca because fish must effectively absorb and conserve phosphate in both FW and SW environments. Dietary P concentration is a major regulator of P metabolism in fish [355]. The amount of phosphate absorbed from the food is affected by the level of phosphate in the blood [6]. The serum concentration of phosphate and the total body content of phosphate are highly regulated and movement into cells is mediated by sodium–phosphate co-transporters [356]. 

Information on the endocrine regulation of P homeostasis in fish is limited. The hormones involved in phosphate regulation include ST, prolactin and parathyroid-like hormones (Pth1h). With the rise in serum Ca, stanniocalcin is secreted by the corpuscles of Stannius to inhibit gill and intestinal Ca transport and to promote P reabsorption in the kidney to maintain normal physiological serum Ca and P levels [357,358]. Parathyroid hormone-like hormones, Pth3 and Pth4, play an important role in functions related to P and bone mineral homeostasis [359,360]. It is also not clear whether the effect is mediated by the vitamin D metabolites as happens in terrestrial vertebrates [361]. Although intraperitoneal injection of vitamin D metabolites influences P homeostasis [362], dietary intake of cholecalciferol had no clear effect on P absorption and retention in rainbow trout [363]. Fjelldal et al. [364] observed that when reared under continuous light, low-P diets affected their plasma 25(OH)D_3_ concentration and normal bone development, indicating a need for an optimum level of dietary P. Low-P diet affected bone osteoblast and osteoclast activity and plasma 1,25(OH)_2_D_3_ levels, whereas the photoperiod had an effect on bone osteoclast activity and plasma 25(OH)D_3_ level.

#### 3.1.1. Requirement

The Ca requirement of fish is affected by dietary factors (e.g., bioavailability, P level), uptake from water and species differences [3,365]. Generally, a large part of the Ca requirement of most fish is met by its absorption through gills in FW and by drinking SW. A low concentration of calcium (0.34 % or less) is required in the diet of carp, red sea bream, striped bass, tilapia, catfish and chum salmon [366,367,368,369,370,371]. Catfish and tilapia reared in water with a low calcium concentration (< 1 mg Ca L^−1^) required 0.45% and 0.7% calcium in the diet, respectively [372,373]. Atlantic salmon absorb Ca from SW, thus making dietary supplementation unnecessary [225]. There is a relatively low requirement (0.1–0.25%) of Ca for farmed marine fish [3]. Other details related to Ca utilization and requirements of certain fish species have been reviewed by Hossain and Yoshimatsu [365] and Lall [96].

The P requirement (g/100g) of a wide range of fish species reared in fresh, brackish and SW have been reported. Requirement in FW: Atlantic salmon, 0.6–1.0 [374,375,376]; rainbow trout, 0.34–0.8 [367,377,378]; chum salmon, 0.5-0.6 [379]; channel catfish, 0.33–0.8 [370,380,381]; milkfish, 0.85 [382]; blue tilapia, 0.5 [373]; Nile tilapia, 0.65-0.86 [383,384]; common carp, 0.6-0.7 [367]; gibel carp, 0.67–1.07 [385]; grass carp, 0.85 [386]; crucian carp, 0.78–0.83 [387]; stinging catfish, 0.9–1.1 [64]; African giant catfish, 1.23 [388]; walking catfish, 0.58–0.73 [389]; Chinese sucker, 0.83–0.86 [390]; hybrid striped bass, 0.5 [391]; snakehead, 0.96 [392]; tambaqui, 0.7 [393]; Japanese eel, 0.29 [63]. Requirement in brackish water (salinity, 5–6 0/00): red drum, 0.86 [394]. Requirement in SW: Atlantic salmon, 0.6 [225]; red sea bream, 0.68 [395]; gilthead seabream, 0.75 [396]; black seabream, 0.55 [397]; haddock, 0.96 [398]; Japanese seabass, 0.86–0.90 [399]; European seabass, 0.65 [400]; orange spotted grouper, 1.09 [401]; yellow croaker, 0.89–0.91 [402]; Japanese flounder, 0.6–1.5 [403,404,405]. 

The requirement estimates mentioned above are mainly based on studies undertaken with juvenile fish and there were differences in dietary sources of P and their bioavailability, fish size and response criteria selected. Antony Jesu Prabhu et al. [406] used a meta-analysis approach to estimate the P requirements based on published information on P utilization for 40 different fish species where requirement values were determined using differences in the response criteria. They found that requirements based on weight gain and P concentrations of the whole body and vertebrae were 0.35, 0.47 and 0.52 g available P/100 g diet (dry matter basis), respectively. Some of these estimates differed from the NRC recommendation on P requirements (g/100g) for major farmed fish species: Atlantic salmon, 0.8; rainbow trout, 0.7; Pacific salmon, 0.6; channel catfish, 0.33; common carp, 0.7; Tilapia sp., 0.4; hybrid striped bass, 0.5; red drum, 0.8; European seabass, 0.65; Japanese flounder, 0.6 P/100 g diet. Obviously, there is a need to better define the P requirements of fish taking into account the stage of development, bioavailability of P from the test diet and feed supplement used to fortify the experimental diets. Studies conducted on other animals show that the maximum growth rates are not necessarily adequate for maximum bone mineralization and they may need higher levels of P in their diet [407]. 

#### 3.1.2. Deficiency

Calcium deficiency has not been detected in carp and catfish in FW [367,380] or in Atlantic salmon in SW [225] fed low-Ca diets. Generally, uptake of Ca from water and absorption from dietary feed ingredients supplies sufficient calcium to meet the requirements of most finfish. Studies conducted on the P requirements of several fish species have shown reduced weight gain, feed utilization and bone mineralization when the P contents of the diets were low, with certain exceptions: gibel carp [385,394], red drum [394], gilthead bream [396] and European seabass [400]. Other P deficiency signs experimentally produced in fish included increases in liver or body fat, reduced blood phosphate levels and poor mineralization of scales. Fish fed either a mineral-deficient or low-P diet mobilize minerals including Ca and P to maintain normal physiological functions [96] and fish scales appear to be the most sensitive indicator of P deficiency in young fish [376,408]. The causes of low dietary P-induced skeletal deformities in salmonids and certain marine fish have been the subject of intensive research and several reviews have been published in this area [30,409,410,411]. Common skeletal deformities with low intake of P include curved spines and soft bones in Atlantic salmon [411], cephalic deformities in the frontal bones of common carp [367] and compressed vertebral bodies resulting in scoliosis in haddock [398] and halibut [410]. Recent studies of bone matrix mineralization in Atlantic salmon have revealed mobilization of minerals during low intake of dietary P and that the normal growth of non-mineralized bone does not fully explain the biochemical mechanism involved in bone P metabolism [412,413,414,415]. Several nutrients support skeletal growth including bone and scale matrix mineralization and the interactions between P and other nutrients in skeletal tissue biomineralization are poorly understood [30]. 

#### 3.1.3. Bioavailability 

The bioavailability of P to fish differs markedly among feed ingredients and inorganic P supplements as well as among other dietary factors (e.g., chemical form, digestibility of diet, particle size, interaction with other nutrients, feed processing and water chemistry) [3,416]. Most of the P in cereal grains and oilseed meals present in the form of an organic complex, phytic acid or phytate (myo-inositol 1, 2, 3, 4, 5, 6-hexakis dihydrogen phosphate) is not available to fish because their intestinal tract does not have sufficient extracellular phytase like other non-ruminant vertebrates. Phytate depresses protein and amino acid digestibility and utilization efficiency in fish and other higher animals. Phytate interactions with proteins are also pH dependent [417]. Several reviews have considered various aspects of phytates in fish nutrition and potential strategies to improve the bioavailability of phytates in feed ingredients of plant origin [271,418,419,420]. The hydrolysis of phytic acid can be achieved by enzymatic and non-enzymatic degradation. Development of phytases and optimization of their catalytic features has been a promising strategy for efficient reduction of phytate in animal feeds. Phytate also forms complexes with lipid and derivatives along with other nutrients [421] but this aspect of chelate formation has not been investigated in fish. The preferable method of phytase application in feeds is coating a liquid form of the enzyme after extrusion and drying which prevents loss of enzyme activity during processing [422]. 

Phosphorus in fish meal is mainly in the form of an insoluble Ca–P complex, hydroxyapatite, which varies with the source of fish used and its bioavailability varies among fish species [2,3]. Acid hydrolysis of fish bone by-products appears to increase P availability of the hydroxyapatite in bone [423]. There are significant differences in the availability of P from a variety of inorganic salts: the more soluble the salt, the higher the availability of P, thus P is more readily available from mono- or di-calcium phosphates than from tri-calcium phosphate [2,3]. Salmonids utilize P present in fish meal more efficiently than carp, tilapia and channel catfish [370,424,425,426].

### 3.2. Magnesium 

Magnesium is a critical intracellular divalent cation that plays an essential physiological role in many functions in the body. It forms a key complex with ATP and plays a key role in many important biological processes such as protein synthesis, cell replication and energy metabolism. Magnesium is a regulator of ion channels, an important intracellular signaling molecule, involved in nerve conduction, muscle contraction and potassium transport, and is a modulator of oxidative phosphorylation. Extracellular Mg is vital to normal nerve conduction, muscle function and skeletal tissue metabolism. It plays an important role in the respiratory adaptation of FW fish [427]. The major proportion (50–70%) of Mg in the body of fish is located in skeletal tissues and scales [428]. The remainder is found within the cells of soft tissues. In muscle, it comprises approximately 20% of the total body Mg pool [137].

Dietary Mg is considered the main source for growth and development of fish [6,429]. When dietary Mg concentration was low in FW, part of its requirement was met by uptake from water via the gills [430,431]. However, in most fish species studied, Mg uptake from FW was insufficient to meet their dietary Mg requirement [3,6]. There is some evidence that excess Mg is excreted renally by fish in FW [432]. In SW, fish absorb Mg by drinking [433] and the major part of the Mg requirement of Atlantic salmon and other marine fish could be met by absorption of SW [225].

#### 3.2.1. Requirement

The magnesium requirements of most farmed fish species range from 0.4 to 0.6 g kg^−1^ diet [3,6,96]. Hybrid tilapia showed Mg requirements of 0.2 and 0.02% in FW and SW, respectively. No requirement of Mg for yellow croaker and red mullet has been reported [434]. A meta-analysis of Mg requirements reported for several fish species showed relatively close estimates for the Mg requirement (g kg^−1^ diet) based on the following parameters: weight gain, 0.34; whole body Mg, 0.49; vertebrae Mg, 0.42; plasma Mg, 0.5 [105]. In SW, Mg requirement for Atlantic salmon and red seabream was not observed [225,435]. Unlike terrestrial animals, the Mg requirement of rainbow trout was not influenced by an increase in dietary Ca and P levels [137]. 

#### 3.2.2. Deficiency

Magnesium is mobilized from bones and scales when dietary Mg intake is low [429]. Deficiency of Mg in carp, catfish, hybrid tilapia, eel and rainbow trout may include one or more of the following signs: anorexia, reduced growth, sluggishness, high mortality and reduced magnesium content [3,30]. In rainbow trout, Mg deficiency also causes calcinosis of the kidney, vertebral deformity and degeneration of muscle fibers and epithelial cells of the pyloric cecum and gill filaments [429,436]. A low concentration of Mg in water reduced the Mn concentration of eggs in carp, which reduced the hatchability of eggs and survival of offspring and also caused deformities and tissue necrosis [437].

#### 3.2.3. Bioavailability

Information on Mg bioavailability from feed ingredients and inorganic supplements for fish is scarce. Generally, natural feed ingredients of plant and animal origin contain moderate levels of Mg. Inorganic Mg feed supplements include magnesium sulfate, magnesium chloride, magnesium oxide and magnesium acetate. Magnesium sulfate is more water soluble than magnesium oxide and therefore more available for absorption. Magnesium present as Mg acetate was more efficiently used by tilapia than either Mg oxide or sulfate [438]. 

## 4. Concluding Remarks

Many gaps exist in the knowledge of mineral nutrition of fish and shrimp related to their dietary requirements, physiological functions, absorption from the gastrointestinal tract and bioavailability from feed ingredients. Information on animal and human nutrition has been useful to confirm the biochemical functions of certain inorganic elements including skeletal tissue metabolism, cellular respiration, oxygen transport and regulation of acid–base equilibrium as well as important components of hormones, enzymes and enzyme activators. Extensive research on farm animals has demonstrated that mineral requirements differ at various stages of their production cycle, certain trace elements play important roles in immune functions and disease prevention and application of specific methodologies are useful to predict the bioavailability of minerals from feed ingredients; however, the research in these areas on fish is limited. Another issue specific to aquatic animals is that there is a need to consider the impact of waterborne minerals from both the nutritional and environmental points of view.

Aquaculture is now the fastest growing food production system globally with many new challenges to address the nutritional problems of more than 40 major farmed fish species. Early studies on mineral nutrition were conducted on salmonids and some warm water fishes using semi-purified diets and trace element supplements of high bioavailability. Most known mineral requirements (Ca, P, Mg, Cu, Zn, Mn, Se) were determined for young fish. The NRC [2,3] requirement values for certain minerals for about 10 fish species have been used as guidelines and as a starting point to establish recommendation allowances for new fish species. Many studies were short term and gave little consideration to the dietary intake or mineral status prior to the experimental period and to the effect that the previous diet may have on body stores at the commencement of the study. Some minerals take longer, depending on the water temperature, to reach a steady state following a change in their dietary intake. A shift from the use of fish meal as a major source of protein and minerals in feeds to proteins of plant origin and land animal products now requires better assessment of mineral bioavailability for improving feed formulation more precisely. There are also new trace element supplements available which require proper assessment of their rate of absorption and potential impact on fish performance.

As an animal’s requirement for any nutrient is affected by many factors, limited data on nutrient requirement values (e.g., NRC [3]) should not be regarded as fixed quantities. Instead of solely relying on these values, changes in animal performance with alterations in nutrient intake should be determined as dynamic responses, to derive requirement estimates that are appropriate to the particular fish species under different culture conditions and dietary regimes. This should start with the units of expression of data on mineral requirements. Meta-analyses of existing data on requirements provide some general guidelines on recommendations. Factorial models are now being applied to estimate nutrient requirements and metabolism of farmed animals and fish; however, additional reliable new data on mineral requirements of major farmed fish species are needed to generate reliable information for use in feed formulation.

## Figures and Tables

**Table 1:** Iron and copper requirement of fish.

Fish Species	Copper ^a^			Iron ^e^		
	mg kg^−1^	Main Response Criteria	Reference	mg kg^−1^	Main Response Criteria	Reference
Atlantic salmon	5–10	Liver Cu	[50]	60–100	H ^f^, liver Fe	[51,52]
				60	Weight gain, H ^f^, Liver Fe	[52]
Rainbow trout	3	Body, vertebral, liver Cu	[53]			
Coho salmon	5.1–5.5	WG, body, vertebrae Cu	[48]			
Channel catfish	5	Liver Cu–Zn SOD	[54]	30	WG, H ^f^	[55]
Yellow catfish	3.1–4.2	WG ^b^, Cu retention	[56]	55.7		[57]
Common carp	3 ^c^	Body, vertebral, liver Cu	[53]	147.4	Serum Fe	[58]
Gibel carp				202	Hematocrit, liver Fe	[59]
Grass carp	4.7–5	WG, plasma ceruplasmin activity	[60]			
Hybrid tilapia	4	WG, body Cu retention	[61]	150–160 ^g^ 85 ^e^	Weight gain, hemoglobin, liver Fe	[62]
Japanese eel				170		[63]
Asian stinging catfish	5.2–5.7	WG, plasma ceruplasmin activity	[64]			
Russian sturgeon	7–8	WG, whole body Cu, liver Cu–Zn SOD, serum ceruloplasmin activity	[65]			
Red sea bream				150		[66]
Tongue sole	11–12	WG, serum Cu–Zn SOD activity	[67]			
Malabar grouper	4–6	WG, liver Cu–Zn SOD activity, body Cu retention	[68]	100 ^h^	Liver Fe	[69]
	2–3 ^d^	WG, liver Cu–Zn SOD activity, body Cu retention	[70]			
Yellow croaker	3.4–7	Serum Cu–Zn SOD activity, body and vertebral Cu	[71]			
Cobia				80.5–94.7 ^e^ 71.3–75.1 ^i^	WG, serum catalase activity	[72]

^a^ Unless specified, CuSO_4_·5H_2_O used as Cu supplement; ^b^ WG = weight gain; ^c^ CuCl_2_ used as Cu supplement; ^d^ copper peptide used as Cu supplement; ^e^ unless specified, FeSO_4_·6H_2_O used as Fe supplement; ^f^ hematology; ^g^ ferric citrate used as Fe supplement; ^h^ requirement for orange-spotted grouper (*Epinephelus coioides*); ^i^ iron methionine used as Fe supplement.

**Table 2:** Manganese requirements of certain fish ^a^.

Fish Species	mg kg^−1^	Main Response Criteria	Reference
Atlantic salmon	15 7.5–10.5	Body and vertebral Mn Body Mn	[127] [128]
Rainbow trout	12–13	WG ^b^	[53]
Channel catfish	2.4	WG	[129]
Yellow catfish	5.5–6.4	WG, vertebral Mn, liver Mn-SOD ^c^	[130]
Common carp	12–13	Growth rate	[53]
Gibel carp	13.8	WG, body and vertebral Mn	[131]
Hybrid tilapia	7	Body Mn, liver Mn-SOD	[132]
Grouper ^d^	15	Body and vertebral Mn	[123]
Yellow croaker	16.4	Growth rate, liver Mn-SOD	[133]
Cobia	21.7–24.9 10.5–15.4 ^e^	WG, body and vertebral Mn Specific growth rate, liver Mn-SOD	[134] [125]

^a^ Unless specified, MnSO_4_·H_2_O used as Mn supplement; ^b^ weight gain; ^c^ liver Mn-SOD activity; ^d^ orange spotted grouper (*Epinephelus coiodes*); ^e^ MnSO_4_·H_2_O, manganese glycine and manganese 2-hydroxy-4-(methylthio)butyrate showed Mn requirements of 15.4, 11.2 and 10.5 mg kg^−1^, respectively.

**Table 3:** Selenium requirements of certain fish.

Fish Species	Requirement, mg kg^−1^	Selenium Source	Main Response Criteria	Reference
Atlantic salmon	0.27 ^a^(0.65)	Na_2_SeO_3_ or SeMet ^b^	Body and tissue Se	[169]
Rainbow trout	0.15–0.38	Na_2_SeO_3_	Plasma GPx	[146]
Coho salmon	0.39–0.43	Na_2_SeO_3_	WG, whole body, liver Se,	[168]
Channel catfish	0.25 0.28, 0.17 0.09, 0.12 0.11, 0.12	Na_2_SeO_3_ Na_2_SeO_3_ SeMet Se-yeast	Liver and plasma GPx WG ^c^, GPx WG, GPx WG, GPx	[170] [171]
Gibel carp	1.18 0.73–1.19	SeMet SeMet	WG, liver GPx, tissue Se Liver Se, liver SOD, T-AOC	[172] [173]
Nile tilapia	0.57	SeMet	WG, liver GPx	[174]
Largemouth bass	1.60–1.85	Na_2_SeO_3_	Liver GPx	[175]
Gilthead sea bream	0.94	Na_2_SeO_3_	Growth, liver Se	[176]
Black sea bream	0.86	Se-polysaccharide ^d^	Liver SOD and GPx	[177]
Malabar grouper	0.7 0.9 0.98	SeMet SeMet Na_2_SeO_3_	WG, Se retention WG, flesh Se	[178] [179]
Cobia	0.8	SeMet	Liver and serum GPx, whole body Se	[180]

^a^ Based on available Se; ^b^ Se-methionine used as Se supplement; ^c^ weight gain; ^d^ Se-polysachharide used as Se supplement.

**Table 4:** Zinc requirements of certain fish ^a^.

Fish Species	Requirement, mg kg^−1^	Main Response Criteria	Reference
Atlantic salmon	37–67	Body and serum Zn	[230]
Rainbow trout	15–30 30.1	WG ^b^, vertebral Zn WG	[231] [232]
Channel catfish	20	WG, vertebral Zn	[233]
Yellow catfish	17.1–20.9	WG, PER ^c^	[234]
Common carp	15	WG, vertebral Zn	[235]
Jian carp	43.2–48.7 ^d^	WG, serum Zn	[236]
Grass carp	55	WG, whole body, vertebral, scale and tissue Zn	[237]
Indian major carp	47.8–52.9	WG, vertebral, scale serum and liver Zn	[238]
Hybrid tilapia	26–29 105–115 ^e^	WG, whole body Zn WG, whole body and plasma Zn	[239] [240]
Blue tilapia	20	Scale and vertebral Zn	[241]
Nile tilapia	30 37.2–52.1	WG, vertebral and serum Zn WG, bone Zn	[242] [243]
Russian sturgeon	28.2–34.6	WG and liver Zn	[244]
Red drum	20	WG, serum and bone Zn	[245]
Blunt snout sea bream	52.1 ^f^, 86.2 ^f^	WG, whole body Zn	[246]
Malabar grouper	28.9–33.7	WG, vertebral and scale Zn	[247]
Cobia	42.9	WG, vertebral Zn	[248]

^a^ Unless specified, ZnSO_4_·7H_2_O used as Zn supplement; ^b^ weight gain; ^c^ protein efficiency ratio; ^d^ zinc lactate (C_6_H_10_O_6_Zn); ^e^ diet based on soybean meal; ^f^ requirements based on weight gain and whole body Zn, respectively.

## Data Availability

Not applicable.
