# Correction: Lall, S.P.; Kaushik, S.J. Nutrition and Metabolism of Minerals in Fish. *Animals* 2021, *11*, 2711

**DOI:** 10.3390/ani11123510

**Published:** 2021-12-09

**Authors:** Santosh P. Lall, Sadasivam J. Kaushik

**Affiliations:** 1 National Research Council of Canada, Halifax, NS B3H 3Z1, Canada; 2 Retd. INRA, 64310 St Pée sur Nivelle, France; 3 Ecoaqua Institute, Universidad de Las Palmas de Gran Canaria, 35214 Las Palmas, Spain

The authors found some omissions and errors in the original paper [1].

Below are provided the full details of the changes in the text and in Table 1.

The authors apologize for any inconvenience caused and state that the scientific conclusions are unaffected. The original publication has been updated.

## 1. Error in Table 1

In the original publication, there was an omission in Table 1 as published. In this Table 1, on line 9, “Gibel carp” should be added. The corrected Table 1 appears below. 

## 2. Text Corrections

On page 4, Section 2.1, first paragraph, line 8, the word “monooxygenase” should be revised to “monoxygenase”.

On page 7, Section 2.1.4, first paragraph, line 13, “and the author contend” should be revised to “suggest”.

On page 8, Section 2.2.2, second paragraph, line 1, the citation [47] should be removed.

On page 14, Section 2.4.4, second paragraph, line 4, the citation [189] should be changed to [202].

On page 16, Section 2.5, fourth paragraph, line 2, the citation [18] should be changed to [19].

On page 17, Section 2.5.3, second paragraph, line 8, the citation [258] should be added to [259].

On page 20. Section 2.8, first paragraph, line 10, the citation [298] should be changed to [324].

On page 21, in Section 2.11, first paragraph, line 3, the citation [46] should be changed to [47].

On page 21, in Section 2.11, first paragraph, line 11, the citation [313] should be changed to [338].

On page 21, in Section 2.11, first paragraph, line 15, the citation [313] should be changed to [47].

On page 21, in Section 2.11, second paragraph, line 1, the citations [319,320] should be removed.

On page 21, in Section 2.11, second paragraph, line 3, the citations [314,315] should be changed to [342,343].

On page 21, in Section 2.11, second paragraph, line 7, the citation [342] should be changed to [344].

On page 21, in Section 2.11, second paragraph, line 8, the citation [201] should be changed to [212].

On page 21, in Section 2.11, second paragraph, line 13, the citation [343] should be changed to [345].

On page 22, Section 3.1, first paragraph, line 3, citation [344] should be changed to [346].

On page 22, Section 3.1, first paragraph, line 9, citation [345] should be changed to [347].

On page 22, Section 3.1, first paragraph, line 11, citation [346] should be changed to [348].

On page 22, Section 3.1, second paragraph, line 4, citations [347–349] should be changed to [349–351].

On page 22, Section 3.1, second paragraph, last line, citations [326,350] should be changed to [328,352–354]. 

On page 22, Section 3.1, third paragraph, line 8, citation [353] should be changed to [355].

On page 22, Section 3.1, third paragraph, last line, citation [354] should be changed to [356].

On page 22, Section 3.1, fourth paragraph, line 5, citations [355,356] should be changed to [357,358].

On page 22, Section 3.1, fourth paragraph, line 7, citations [357,358] should be changed to [359,360].

On page 22, Section 3.1, fourth paragraph, line 8, citation [359] should be changed to [361].

On page 22, Section 3.1, fourth paragraph, line 9, citation [360] should be changed to [362].

On page 23, Section 3.1, fourth paragraph, line 10,11, citation [361] should be changed to [363] and citation [362] should be changed to [364].

On page 23, Section 3.1.1, first paragraph, line 2, citations [3,363] should be changed to [3,365].

On page 23, Section 3.1.1, first paragraph, line 5, the citations [364–369] should be changed to [366–371].

On page 23, Section 3.1.1, first paragraph, line 7, the citations [370,371] should be changed to [372,373].

On page 23, Section 3.1.1, first paragraph, line 10, the citation [363] should be changed to [365].

On page 23, Section 3.1.1, second paragraph, line 2, the citations [372–374] should be changed to [374–376].

On page 23, Section 3.1.1, second paragraph, line 3, the citations [365,375,376] should be changed to [367,377,378]; the citation for chum salmon [377] should be changed to [379].

On page 23, Section 3.1.1, second paragraph, line 4, the citations [368,378,379] should be changed to [370,380,381]; the citation [380] should be changed to [382] and that for [371] should be changed to [373]; the citations [381,382] should be changed to [383,384].

On page 23, Section 3.1.1, second paragraph, line 5, that for [365] should be changed to [367] and the citation [383] should be changed to [385], that for [384] should be changed to [386].

On page 23, Section 3.1.1, second paragraph, line 6, [385] should be changed to [387]; the citation [386] should be changed to [388].

On page 23, Section 3.1.1, second paragraph, line 7, [387] should be changed to [389]; the citation [388] should be changed to [390]; the citation [389] should be changed to [391].

On page 23, Section 3.1.1, second paragraph, line 8, [390] should be changed to [392]; the citation [391] should be changed to [393].

On page 23, Section 3.1.1, second paragraph, line 9, the citation [392] should be changed to [394].

On page 23, Section 3.1.1, second paragraph, line 11, the citation for “…Atlantic salmon, 0.6 (Lall and Bishop, 1977)” should be changed as “…Atlantic salmon, 0.6 [225]”.

On page 23, Section 3.1.1, second paragraph, line 11, the citation [393] should be changed to [395] and that for [394] should be changed to [396].

On page 23, Section 3.1.1, second paragraph, line 12, the citation [395] should be changed to [397], that for [396] should be changed to [398] and that for [397] should be changed to [399].

On page 23, Section 3.1.1, second paragraph, line 13, the citation [398] should be changed to [400], that for [399] should be changed to [401] and that for [400] should be changed to [402].

On page 23, Section 3.1.1, second paragraph, last line, the citations [401–403] should be changed to [403–405].

On page 23, Section 3.1.1, third paragraph, line 3, the citation [404] should be changed to [406].

On page 23, Section 3.1.1, third paragraph, the last line, the citation [161] should be changed to [407].

On page 23, Section 3.1.2, first paragraph, line 1, the citations [365,378] should be changed to [367,380].

On page 23, Section 3.1.2, first paragraph, line 6, the citations [383,392] should be changed to [385,394].

On page 24, Section 3.1.2, first paragraph, line 7, the citation [392] should be changed to [394], that of [394] should be changed to [396] and that for [398] should be changed to [400].

On page 24, Section 3.1.2, first paragraph, line 12, the citations [374,405] should be changed to [376,408].

On page 24, Section 3.1.2, first paragraph, line 14, the citations [30,406–408] should be changed to [30,409–411].

On page 24, Section 3.1.2, first paragraph, line 15, the citation [408] should be changed to [411] 

On page 24, Section 3.1.2, first paragraph, line 16, the citation [365] should be changed to [367].

On page 24, Section 3.1.2, first paragraph, line 17, the citation [396] should be changed to [398]; the citation [407] should be changed to [410].

On page 24, Section 3.1.2, first paragraph, line 20, the citations [387–390] should be changed to [412–415].

On page 24, Section 3.1.3, first paragraph, line 3, the citations [3,409] should be changed to [3,416].

On page 24, Section 3.1.3, first paragraph, line 9, the citation [410] should be changed to [417].

On page 24, Section 3.1.3, first paragraph, line 11, the citations [271,411–413] should be changed to [271,418–420].

On page 24, Section 3.1.3, first paragraph, line 14, the citation [414] should be changed to [421].

On page 24, Section 3.1.3, first paragraph, last line, the citation [415] should be changed to [422].

On page 24, Section 3.1.3, second paragraph, line 4, the citation [416] should be changed to [423].

On page 24, Section 3.1.3, second paragraph, last line, the citations [368,417–419] should be changed to [370,424–426].

On page 24, Section 3.2, first paragraph, line 8, the citation [420] should be changed to [427].

On page 24, Section 3.2, first paragraph, line 9, the citation [421] should be changed to [428].

On page 25, second paragraph, line 1, the citations [6,422] should be changed to [6,429].

On page 25, second paragraph, line 3, the citations [423,424] should be changed to [430,431].

On page 25, second paragraph, line 5, the citation [425] should be changed to [432]; the citation [426] should be changed to [433].

On page 25, Section 3.2.1, line 3, the citation [427] should be changed to [434].

On page 25, Section 3.2.1, line 7, the citations [225,428] should be changed to [225,435].

On page 25, Section 3.2.2, line 1, the citation [422] should be changed to [429].

On page 25, Section 3.2.2, line 6, the citations [422,429] should be changed to [429,436].

On page 25, Section 3.2.2, last line, the citation [430] should be changed to [437].

On page 25, Section 3.2.3, last line, the citation [431] should be changed to [438].

The authors sincerely apologize for any inconvenience caused and state that the scientific conclusions are unaffected. The original publication has also been updated.

## 3. References

Some references (Refs. [342,343,407,412,413,414,415]) have been added and the associated numbering has been updated accordingly.

342. Cockell, K.A.; Hilton, J.W.; Bettger, W.J. Chronic toxicity of dietary disodium arsenate heptahydrate to juvenile rainbow trout (*Oncorhynchus mykiss*). *Arch. Environ. Contam. Toxicol* **1991**, *21*, 518–527.
343. Erickson, R.J.; Mount, D.R.; Highland, T.L.; Russell Hockett, J.; Jenson, C.T. The relative importance of waterborne and dietborne arsenic exposure on survival and growth of juvenile rainbow trout. *Aquat. Toxicol* **2011**, *104*, 108–115.
407. National-Research-Council, N. *Nutrient Requirements of Swine*; The National Academies Press: Washington, DC, USA, 2012.
412. Drábiková, L.; Fjelldal, P.G.; De Clercq, A.M.N.; Morken, T.; McGurk, C.; Witten, P.E. Vertebral column adaptations in juvenile Atlantic salmon *Salmo salar*, L. as a response to dietary phosphorus. *Aquaculture* **2021**, *541*, 736776, doi:10.1016/j.aquaculture.2021.736776.
413. Fjelldal, P.G.; Hansen, T.; Breck, O.; Ørnsrud, R.; Lock, E.J.; Waagbø, R.; Wargelius, A.; Eckhard Witten, P. Vertebral deformities in farmed Atlantic salmon (*Salmo salar* L.) – etiology and pathology. *J. Appl. Ichthyol.* **2012**, *28*, 433–440, doi:10.1111/j.1439-0426.2012.01980.x.
414. Witten, P.E.; Fjelldal, P.G.; Huysseune, A.; McGurk, C.; Obach, A.; Owen, M.A.G. Bone without minerals and its secondary mineralization in Atlantic salmon (*Salmo salar*): The recovery from phosphorus deficiency. *J. Exp. Biol.* **2019**, *222*, 188763.
415. Witten, P.E.; Owen, M.A.; Fontanillas, R.; Soenens, M.; McGurk, C.; Obach, A. A primary phosphorus-deficient skeletal phenotype in juvenile Atlantic salmon *Salmo salar*: The uncoupling of bone formation and mineralization. *J. Fish Biol.* **2016**, *88*, 690–708, doi:10.1111/jfb.12870.

## Figures and Tables

**Table 1:** Iron and copper requirement of fish.

Fish Species	Copper ^a^			Iron ^e^		
	mg kg^−1^	Main Response Criteria	Reference	mg kg^−1^	Main Response Criteria	Reference
Atlantic salmon	5–10	Liver Cu	[50]	60–100	H ^f^, liver Fe	[51,52]
				60	Weight gain, H ^f^, Liver Fe	[52]
Rainbow trout	3	Body, vertebral, liver Cu	[53]			
Coho salmon	5.1–5.5	WG, body, vertebrae Cu	[48]			
Channel catfish	5	Liver Cu–Zn SOD	[54]	30	WG, H ^f^	[55]
Yellow catfish	3.1–4.2	WG ^b^, Cu retention	[56]	55.7		[57]
Common carp	3 ^c^	Body, vertebral, liver Cu	[53]	147.4	Serum Fe	[58]
Gibel carp				202	Hematocrit, liver Fe	[59]
Grass carp	4.7–5	WG, plasma ceruplasmin activity	[60]			
Hybrid tilapia	4	WG, body Cu retention	[61]	150–160 ^g^ 85 ^e^	Weight gain, hemoglobin, liver Fe	[62]
Japanese eel				170		[63]
Asian stinging catfish	5.2–5.7	WG, plasma ceruplasmin activity	[64]			
Russian sturgeon	7–8	WG, whole body Cu, liver Cu–Zn SOD, serum ceruloplasmin activity	[65]			
Red sea bream				150		[66]
Tongue sole	11–12	WG, serum Cu–Zn SOD activity	[67]			
Malabar grouper	4–6	WG, liver Cu–Zn SOD activity, body Cu retention	[68]	100 ^h^	Liver Fe	[69]
	2–3 ^d^	WG, liver Cu–Zn SOD activity, body Cu retention	[70]			
Yellow croaker	3.4–7	Serum Cu–Zn SOD activity, body and vertebral Cu	[71]			
Cobia				80.5–94.7 ^e^ 71.3–75.1 ^i^	WG, serum catalase activity	[72]

^a^ Unless specified, CuSO_4_·5H_2_O used as Cu supplement; ^b^ WG = weight gain; ^c^ CuCl_2_ used as Cu supplement; ^d^ copper peptide used as Cu supplement; ^e^ unless specified, FeSO_4_·6H_2_O used as Fe supplement; ^f^ hematology; ^g^ ferric citrate used as Fe supplement; ^h^ requirement for orange-spotted grouper (*Epinephelus coioides*); ^i^ iron methionine used as Fe supplement.
